# Correlated evolution between climate and suites of traits along a fast–slow continuum in the radiation of *Protea*

**DOI:** 10.1002/ece3.3773

**Published:** 2018-01-12

**Authors:** Nora Mitchell, Jane E. Carlson, Kent E. Holsinger

**Affiliations:** ^1^ Department of Ecology and Evolutionary Biology University of Connecticut Storrs CT USA; ^2^ Inventory and Monitoring Program Gulf Coast Network National Park Service Lafayette LA USA

**Keywords:** adaptation, adaptive radiation, functional traits, niche, Proteaceae

## Abstract

Evolutionary radiations are responsible for much of Earth's diversity, yet the causes of these radiations are often elusive. Determining the relative roles of adaptation and geographic isolation in diversification is vital to understanding the causes of any radiation, and whether a radiation may be labeled as “adaptive” or not. Across many groups of plants, trait–climate relationships suggest that traits are an important indicator of how plants adapt to different climates. In particular, analyses of plant functional traits in global databases suggest that there is an “economics spectrum” along which combinations of functional traits covary along a fast–slow continuum. We examine evolutionary associations among traits and between trait and climate variables on a strongly supported phylogeny in the iconic plant genus *Protea* to identify correlated evolution of functional traits and the climatic‐niches that species occupy. Results indicate that trait diversification in *Protea* has climate associations along two axes of variation: correlated evolution of plant size with temperature and leaf investment with rainfall. Evidence suggests that traits and climatic‐niches evolve in similar ways, although some of these associations are inconsistent with global patterns on a broader phylogenetic scale. When combined with previous experimental work suggesting that trait–climate associations are adaptive in *Protea*, the results presented here suggest that trait diversification in this radiation is adaptive.

## 1 INTRODUCTION

Evolutionary radiations are responsible for much of the diversity of life on Earth, but only some of them are adaptive. To be regarded as adaptive, diversification within lineages into new species and morphological forms must be associated with diversity in ecological roles (Givnish, 2015; Schluter, 2000). Diversification may occur whenever there is geographic, ecological, or evolutionary opportunity associated with genetic differentiation, reproductive isolation, and ecological divergence (Givnish 2010; Glor, 2010; Simões et al., 2016; Simpson, 1955). The extent to which diversification is driven by adaptive processes and natural selection often remains unclear (Givnish, 1997, 2015), as radiations can also be the by‐product of divergence via geographic isolation associated with stochastic or neutral processes (nonadaptive radiations, Kozak, Weisrock, & Larson, 2006; Rundell & Price, 2009). If divergence within a rapidly diversifying lineage is primarily associated with divergence among related species due to ecological opportunity, such as in Simpson's concept of the “adaptive zone” (Simpson, 1955), it may be classified as an adaptive radiation (Givnish, 2015).

Tracing the simultaneous evolution of individual traits and climate allows us to assess the role that adaptation to climate played in generating trait diversity. If adaptation is important in trait diversification, then evolutionary changes in functional phenotypic traits, those with presumed effects on survival, growth, and reproduction in the context of the abiotic environment (Violle et al., 2007), should be associated with changes in some aspect of the climatic‐niche (although changes in biotic associations may also play a role). In plants, there is a wealth of interest in so‐called economics spectra along a fast–slow continuum, such as the worldwide leaf economics spectrum (Wright et al., 2004), the wood economics spectrum (Chave et al., 2009), and the whole‐plant spectrum (e.g., Edwards, Chatelet, Sack, & Donoghue, 2014; Reich, 2014). A recent global analysis identified two main dimensions of variation in plant traits: one along the leaf economics spectrum and another related to plant size (Díaz et al., 2016). Different suites of traits may be related to different aspects of the climatic‐niche, and the global relationships that exist between traits and climate variables such as precipitation and temperature need to be tested within individual regions and lineages (Messier, Lechowicz, McGill, Violle, & Enquist, 2017; Moles et al., 2014; Wright et al., 2005). Trait–climate associations along the branches of a phylogeny suggest that adaptation plays a role in trait diversification. There is also substantial evidence for integrated trait evolution (phenotypic integration through evolutionary time) in plants (e.g., the worldwide leaf economics spectrum, Wright et al. 2004), so patterns of covariation within traits and within climate variables also need to be identified.

Trait–climate associations can be observed at many spatial and temporal scales. For example, statistical associations between field‐measured traits and environmental parameters provide evidence that contemporary trait differences are associated with important physiological and ecological functions both among and within distantly related genera in South Africa (Mitchell et al., 2015). By themselves, however, such associations do not provide evidence that differentiation in those functions played an important role in evolutionary diversification among species. Moreover, in field‐based studies, observed differences could be the result of phenotypic plasticity rather than differential adaptation, a distinction that can be made only with common‐garden studies (e.g., Givnish & Montgomery, 2014; Mason & Donovan, 2015). Evidence for the adaptive nature of trait–climate associations is strengthened by analyses incorporating evolutionary history. Specifically, assessing associations across a phylogeny requires the use of methods incorporating evolutionary relationships among taxa (such as phylogenetically independent contrasts, Felsenstein, 1985, or phylogenetic generalized least squares, Martins & Hansen, 1997). These approaches provide measures of trait–trait or trait–climate associations while controlling for phylogeny, that is, “evolutionary associations.”

We search for evolutionary trait–climate associations in a species‐rich and morphologically diverse plant lineage located in a biodiversity hotspot characterized by multiple climatic gradients. The genus *Protea* L. (Proteaceae) is a diverse group with 112 known evergreen plant species displaying diversity in growth form (ranging from subshrubs to shrubs and small trees), leaf shape and size, and inflorescence architecture (Rebelo, 2001; Valente et al., 2010). The age of the group is uncertain, but best estimates place the crown age at 5–18 my (Sauquet et al., 2009). The genus has its center of diversity in the Cape Floristic Region (CFR) of South Africa (Valente et al., 2010), a biodiversity hotspot characterized by high levels of species diversity (over 9,000 plant species) and endemism (about 70%, Goldblatt & Manning, 2002) in addition to being particularly threatened by human impacts (Myers, Mittermeier, Mittermeier, da Fonseca, & Kent, 2000). *Protea* is one of the dominant members of the fynbos community, although its range extends into northern and eastern portions of South Africa, Lesotho, Kenya, and central Africa (Rebelo, 2001; Rourke, 1980; Valente et al., 2010).

The extraordinary plant diversity in South Africa has been attributed to several different factors: the topographical complexity of multiple mountain ranges and “sky islands,” sharp changes in soil types, soils that are extremely low in nutrients, steep gradients in temperature and in rainfall amount and seasonality, and the onset of the present‐day climate dated at the Miocene–Pliocene boundary some 10 million years ago (Campbell, 1983; Linder, 2003; Verboom, Bergh, Haiden, Hoffmann, & Britton, 2015; Verboom et al., 2009). This diversity is largely accounted for by high species diversity in just 33 evolutionary radiations (Linder, 2003; Linder & Hardy, 2004; Schnitzler et al., 2011). Although an abundance of work has addressed the extent to which climatic and environmental heterogeneity has driven speciation and radiation throughout the region (e.g., Lamont, He, & Downes, 2013; Latimer, Silander, Rebelo, & Midgley, 2009; Linder, 2003; Verboom et al., 2009, 2015), few systems have linked evidence from population genetics, common‐garden experiments, and evolutionary analyses in this framework.

Previous studies in *Protea* have documented contemporary associations between morphological traits and the environment across the genus (Mitchell et al., 2015). Experimental work within a smaller clade (the white proteas) and a single species (*Protea repens*) have found evidence for genetic differentiation of traits related to the environment consistent with adaptive differentiation in physiology and fitness (Carlson, Adams, & Holsinger, 2015; Carlson, Holsinger, & Prunier, 2011; Prunier, Holsinger, & Carlson, 2012). Here, we expand on these results and ask whether the diversification of traits is correlated with climatic factors across the genus in South Africa. More specifically, we ask:

1. Are there correlations between suites of fast–slow spectrum traits and species' climatic‐niches across *Protea*? How do these compare with global patterns?
2. Have traits and climatic‐niche evolved in similar ways?
3. Are patterns of integrated evolution consistent with patterns of covariation observed in global datasets?

The answers to these questions allow us to identify suites of traits that have correlated evolution with suites of climatic‐niche variables. In answering these questions, we also address how phylogenetic uncertainty and variation in both traits and climatic‐niche values affect our conclusions.

## 2 METHODS

All analyses were carried out in R v3.3.1 (R Core Team 2016), and large‐scale analyses were carried out on the Computational Biology Core Facility of the University of Connecticut. Data are deposited at dryad https://doi.org/10.5061/dryad.h2j0n.

### 2.1 Incorporation of uncertainty

Existing methods for analyzing trait‐by‐trait or trait‐by‐climate associations at evolutionary timescales suffer from two important limitations: (1) They often assume that traits are uniform within species and (2) they usually ignore uncertainty in phylogenetic estimates (but see Huelsenbeck, Rannala, and Masly (2000)). These limitations may be especially important in radiations where diversification has occurred quickly, resulting in soft polytomies and uncertainty in species relationships. The use of Bayesian posterior tree samples or bootstrap replicates can account for some phylogenetic uncertainty (Huelsenbeck & Rannala, 2003), and very recent methods have begun to incorporate this uncertainty into estimates of correlated trait evolution (Caetano & Harmon, 2017), but the role of intraspecific trait variation has typically been neglected. The magnitude of intraspecific trait variation is often quite large, especially in some often‐used plant functional traits (Auger & Shipley, 2013; Carlson et al., 2015; Donovan, Mason, Bowsher, Goolsby, & Ishibashi, 2014). This variation may have large impacts on comparative studies (Garamszegi & Møller, 2010), motivating the modeling of trait variances as well as means in evolutionary studies (Kostikova, Silvestro, Pearman, & Salamin, 2016). These analyses, however, are difficult to carry out on large datasets. We incorporate intraspecific variation in traits, climatic‐niche, and phylogeny throughout our analyses.

### 2.2 Trait measurements

**Figure 1:**
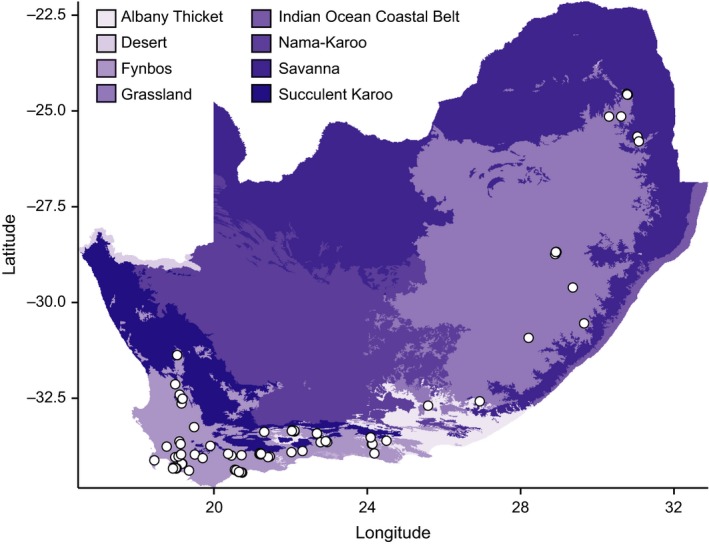
Map of individuals sampled for trait data for this study. Colors correspond to biomes as defined by Mucina and Rutherford (2006). Voucher and latitude/longitude data can be found in Table S1

We measured a suite of traits on plants from 58 different *Protea* species in the field from 2011–2013, including leaf and whole‐plant traits associated with the leaf economics spectrum, as well as those found to be important in previous common‐garden work (Carlson et al., 2011, 2015; Prunier et al., 2012). We incorporated additional traits measured by Carlson et al. (2011) from 2008–2009, resulting in 133 species × site combinations that covered most of the range of *Protea* (Figure 1, average No. of observations per species = 26, range = 1–203). There were 1520 observations, of which <10 percent are complete, by design (See Table S1 for full data). For most populations (species × site combinations), we sampled eight plants for trait measurements, including height and canopy area (estimated from measured orthogonal dimensions of the plant using the formula for an ellipse) and sampled one of the most recently fully expanded leaves per plant. For shrub‐like species, we also harvested wood samples from the previous year's growth for two plants per population. We measured leaf fresh weights and scanned leaves for analysis of length, width, and area in ImageJ (National Institutes of Health, Bethesda, MD, USA). Leaves were then dried and reweighed for dry weights. We did not include saturated weights in results presented here because preliminary analyses from 2011 found a correlation of 0.994 between saturated and fresh weights. For one to two leaves per population, we made stomatal peels on the adaxial side using clear nail varnish and tape that were later analyzed under a light microscope to estimate stomatal size and density. Four leaves per population were analyzed for carbon and nitrogen isotopes at the Stable Light Isotope Laboratory in the Archaeology Department at the University of Cape Town. Wood density was estimated using a water displacement method as dry mass/wet volume (Cornelissen et al., 2003). We combined these data with similar data reported for the white *Protea* clade in Carlson et al. (2011). Final traits used in this analysis include the following: plant height (cm), plant canopy area (cm^2^), leaf mass per area (lma, g/cm^2^; dry leaf mass/fresh leaf area), leaf freshwater content (fwc; [leaf fresh weight–leaf dry weight/leaf dry weight]), leaf length‐to‐width ratio (lwr; leaf length/leaf width), leafarea (cm^2^), stomatal density (sd, stomates/cm^2^), leaf nitrogen per unit mass (nmass; mg/g), leaf ^13^C/^12^C ratio (δ ^13^C), leaf carbon‐to‐nitrogen ratio (cnratio), and wood density (wood, g/cm^3^), Table 1. We natural‐log‐transformed all traits except for δ13C prior to analysis.

**Table 1:** Trait and climate variables examined in this study

Trait	Description
height	Plant height (cm)
canopy	Canopy area (cm^2^)
lma	Leaf mass per area (g/cm^2^)
wood	Wood density
lwr	Leaf length‐to‐width ratio
fwc	Leaf freshwater content (g/*g*_dw_)
sd	Stomatal density (stomates/cm^2^)
nmass	Leaf nitrogen per mass (%)
δ13C	Leaf^13^c:^12^C (‰)
cnratio	Leaf carbon : nitrogen ratio

Climate	Description
mat	Mean annual temperature (°C)
map	Mean annual precipitation (mm)
elev	Elevation (m)
pet	Mean annual potential evapotranspiration (mm)
rfl2 mm	Days with >2 mm rain (days)
rflcv	Interannual coefficient of variation of precipitation (%)
summer_rain	Mean monthly rainfall summed across summer months, December–February (mm)
*t*_max_	Average daily maximum temperature in January (°C)
*t*_min_	average daily minimum temperature in July (°C)
*t*_var_	Maximum–minimum temperature (°C)

Instead of using species means to characterize traits in comparative analyses, we used Bayesian linear models to estimate distributions of trait values within each species, thereby obtaining a distribution that specifies the probability of particular values for each species and is consistent with the observed data. Specifically, we used the stan_glmer (or stan_glm if only one population) function in the R package “rstanarm” (Gabry & Goodrich, 2016) to model values for each log‐transformed trait and each species using an informative prior from a normal distribution (with a mean of the actual trait mean, and standard deviation of the actual standard deviation in trait values) and a site random effect. We do not have information regarding phylogenetic relatedness among populations (sites), so we treat populations as equally related to one another. We ran each model for 5,000 iterations saved 10,000 samples from each posterior distribution. We calculated mean values and the 95% highest posterior density (HPD) intervals (using R/coda) and randomly sampled 100 values per trait per species for use in analyses of trait evolution. The 100 sample values and mean values for each species‐trait combination are reported in Tables S2 and S3, respectively. See Figure S1 for density plots of 100 randomly selected samples per species per phenotypic trait.

### 2.3 Characterizing climatic‐niches

We used Maximum Entropy Modeling (MaxEnt, Phillips and Dudik 2008) to characterize the climatic‐niche for all 58 *Protea* species in our dataset. Latitude and longitude occurrence data for each species were extracted from the Protea Atlas database <http://www.proteaatlas.org.za/>. The occurrence data included 94,715 individual georeferenced records across our species. Climate variables for each georeferenced point were extracted from the South African atlas of agrohydrology and climatology layers (Schulze et al., 2007) at the resolution of 1 by 1 min, or 1.55 by 1.85 km. Sites were grouped together if they were in the same grid cell, and all species observed in that grid cell were recorded. We retained ten variables that capture climatic gradients in the CFR and were used previously in the literature (Table 1): mean annual temperature (mat), average daily minimum temperature in July (*t*_min_), average daily maximum temperature in January (*t*_max_), elevation (elev), mean annual precipitation (map), interannual rainfall variability, measured as the coefficient of variation of mean annual rainfall across years (rflcv), temperature variability measured as maximum–minimum temperature at a site (*t*_var_), mean annual potential evapotranspiration (pet), and two more direct measures of drought: the number of days receiving >2 mm of rain in the three driest months, hence lower values reflect more drought (rfl2 mm), and mean summer rainfall, December–February (summer_rain). Although many of these are highly correlated, we retain them to identify suites of climate variables that are associated with trait variation. All analyses were performed using MaxEnt in R through the package “dismo” (Hijmans, Phillips, Leathwick, & Elith, 2013). For each species in the dataset, we used 90% of the data for training and left 10% for testing. Random pseudoabsences were taken from the extent of South Africa, because species of *Protea* are found in most South African biomes. Details on the MaxEnt sampling and model results can be found in Table S4.

Similar to the trait values, we also incorporated uncertainty into estimates for climate niche variables to obtain mean values and a set of likely values for each species. We used the raw probabilities generated from the MaxEnt models to generate histograms for each species and square‐root transformed climate variables (except for *t*_min_, which was left untransformed) using custom scripts from S.D. Smith (see Evans et al. 2009). These give a distribution of the predicted occupancy profile for each species in each climate variable independent of the other variables. From each of these distributions, we randomly sampled 10,000 observations and then calculated the 95% HPD intervals for each variable and each species in the R package “coda” (Plummer, Best, Cowles, & Vines, 2006). From these 9,500 samples, we calculated means per variable per species (Table S3) and also randomly selected 100 observations for use in downstream analyses (Table S2). Figure S1 has density plots of 100 randomly selected samples per species per niche trait.

### 2.4 Accounting for uncertainty in phylogeny and traits

**Figure 2:**
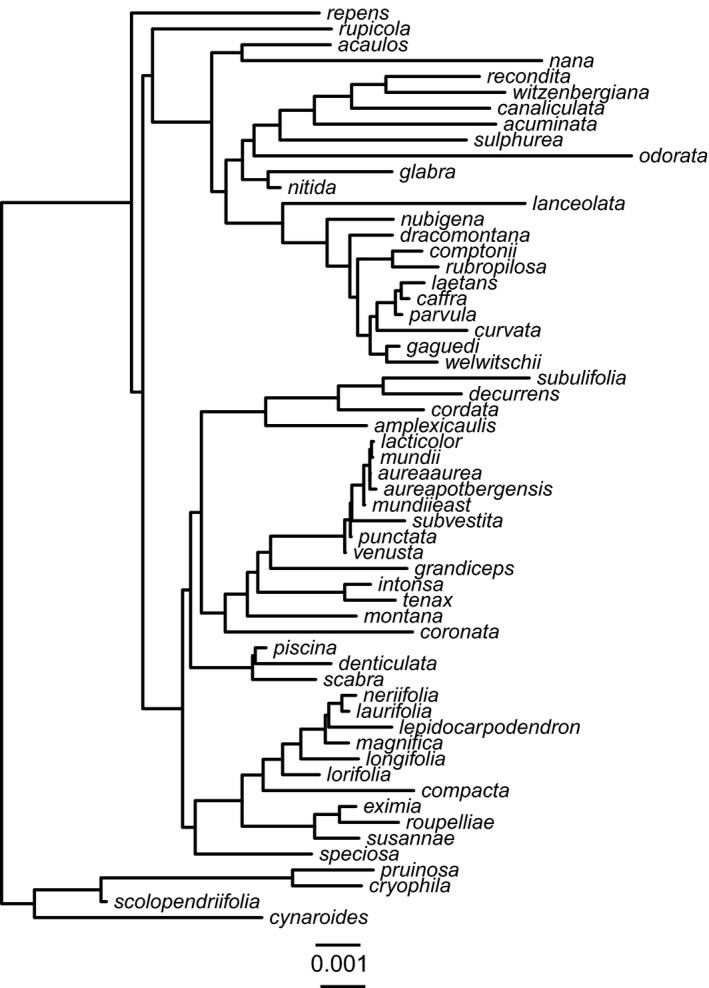
Phylogeny used as the “best” tree generated from ASTRAL‐II in Mitchell et al. (2017). Branches black in color supported by 90% bootstrap support or higher, and branches in gray have <90% bootstrap support. Scale bar corresponds to the number of substitutions per site

It is often difficult to estimate phylogenetic relationships in rapid radiations (Knowles & Chan, 2008). In earlier work, we used an anchored phylogenomics approach (Lemmon, Emme, & Lemmon, 2012) to sequence almost 500 nuclear genes conserved across all angiosperms (Buddenhagen et al., In Review) and built a robust and highly resolved phylogeny for 59 *Protea* species (Mitchell, Lewis, Moriarty Lemmon, Lemmon, & Holsinger, 2017; Buddenhagen et al. In Review). To ensure that our results are robust in the face of both phylogenetic and trait/climate uncertainty, we compared results of analyses using the “best” tree from the program ASTRAL‐II (Figure 2; Mirarab & Warnow, 2015) or 100 bootstrap replicates from the ASTRAL‐II analysis of Mitchell et al. (2017) with either the posterior mean of the trait/climate distribution or 100 random samples from the HPD distribution. We thus have a two‐by‐two table comparing one measure (mean trait on the best tree), 100 measures (mean trait on 100 bootstrap trees; 100 observations of traits on best tree), or 10,000 measures (100 observations of traits on 100 bootstrap trees). From here forward, we refer to datasets as number of trait/climate observations × number of bootstrap trees, organized in order of increasing effort to account for uncertainty (1 × 1, 1 × 100, 100 × 1, 100 × 100). We sort the outputs from 100 × 100 analyses and select the 2.5%, 50%, and 97.5% values to compare with the lower, middle, and upper bounds with the 1 × 1 analyses.

### 2.5 Correlated evolution between traits and climate

We tested for correlations between traits and climate niche variables taking into account evolution using the program BayesTraits (Pagel & Meade, 2007) through R using the wrapper package “btw” (Griffin, 2015). BayesTraits analyzes continuous traits using a PGLS framework under the assumption of Brownian Motion, estimating correlation coefficients and measures of support for correlated evolution between variables. For each set of trees and morphological traits/climate variables, we evaluated a model using the continuous function under MCMC settings, estimating the log marginal likelihood using the stepping stone method (SS, Xie et al. 2010) with 100 stones and 1,000 iterations per stone (increasing the number of stones to 200 or iterations per stone to 10,000 had little effect on estimates, data not shown). We estimated the log Bayes factor (logBF) for the dependent model (allowing correlation between variables) against the independence model (which fixes all correlations to be zero) as twice the difference between the estimated log marginal likelihoods using the formula logBF = 2*(SSdep – SSindep), where SSdep and SSindep are the estimated log marginal likelihoods for the dependent and independent models, respectively. We interpreted comparisons where logBF > 2 as having weak support, logBF > 5 as having moderate support, and logBF > 10 as having strong support. This analysis was performed on all 110 trait–climate combinations for the 1 × 1 and 100 × 100 datasets. Using our logBF > 5 as moderate support in favor of the model with the higher marginal likelihood (Kass & Raftery, 1995), we can account for multiple comparisons by identifying those comparisons in which logBF > 5 +  log(*N*), where *N* is the number of model comparisons performed. For the 100 trait–climate combinations, this corresponds to a logBF > 9.7.

### 2.6 Evolutionary models

Comparisons of models of evolution can help to determine whether phylogenetic signal in traits is the result of phylogenetic niche conservatism. If both traits and climate variables are relatively conserved, then the climatic‐niche may have played a role in trait evolution. If one or the other is not conserved, local or contemporary processes may be more important. A default assumption for trait evolution is that ancestor and descendant species will resemble each other. If phenotypic trait diversification in a radiation is driven by climate, we should expect not only resemblance in traits, but also some degree of niche conservatism, where descendant species occupy climatic‐niches similar to those of their ancestors (Peterson 1999). If, however, phylogenetic similarity in traits is not driven by climate, we may find a lack of niche conservatism.

To determine the evolutionary model that best fits each trait or climatic‐niche variable, we used the fit continuous function in the R package “geiger” (Harmon, Weir, Brock, Glor, & Challenger, 2008) to compare the fit under Brownian motion (BM), Ornstein–Uhlenbeck (OU), and white noise models, setting the bounds for the alpha parameter in the OU model from 0 to 1,000. Model fit was evaluated using AICc scores and Akaike weights. We identify the best fit model using AICc scores using the “best” tree and mean trait/climate value, but also report results incorporating uncertainty across all four datasets (1 × 1, 1 × 100, 100 × 1, and 100 × 100).

### 2.7 Correlated evolution among traits and among climate niche

*A priori* we expect correlations among traits and among climate variables in our dataset. We estimated coefficients taking evolution into account using BayesTraits as above for the 55 trait–trait and 45 climate–climate comparisons. To account for multiple comparisons, we used a Bayes factor cutoff of 9.8 for trait–trait comparisons and 9.6 for climate–climate comparisons. We built separate distance‐based dendrograms for traits and climate variables using the correlation matrices from the 1 × 1 BayesTraits analysis to identify clusters for later analysis.

### 2.8 Supplemental analyses

We performed two additional sets of analyses to ensure that our results are insensitive to important modeling choices. First, to assess the influence of log‐transforming trait and climate data we performed all analyses on the 1 × 1 datasets using untransformed data and found results qualitatively similar to those we obtained using log‐transformed data. Second, to assess the influence of the method of phylogenetic inference we used, we also performed all analyses on the 1 × 1 datasets using the best trees identified using SVDquartets (Chifman & Kubatko, 2014) and RAxML (Stamatakis, 2014) and found results qualitatively similar to those we obtained using ASTRAL‐II.

## 3 RESULTS

### 3.1 Trait–climate correlated evolution

**Figure 3:**
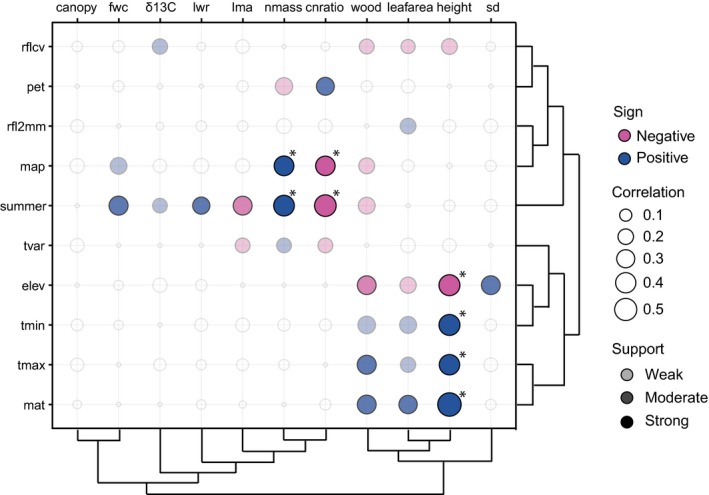
Trait–climate associations for evolutionary analyses. Correlations are either positive (blue) or negative (magenta), vary in strength (size of circle), and have different levels of support indicated by transparency of circle color (weak support: logBF > 2, *p* < .10, most transpaent; moderate support: logBF > 5, *p* < .05, medium transparency; strong support: logBF > 10, *p* < .01, darkest circles). Correlations not supported at any level have no fill (completely transparent). Asterisks indicate correlations that meet the criterion threshold correcting for multiple comparisons. Dendrograms for the evolutionary analyses are based on distance matrices, and order is preserved in the contemporary data to more easily make visual comparisons

We detected correlated evolution between morphological and climatic‐niche traits in only a relatively small number of cases in two main clusters of strongly supported evolutionary associations. These include (1) plant size (leafarea, height, wood density) and its positive association with temperature (mat, *t*_max_, *t*_min_) and negative relationships with rflcv and elev and (2) leaf composition (fwc, δ13C, lwr, nmass, cnratio, lma) with precipitation (summer_rain, map), where higher investment in leaves is found in drier areas (Figure 3). In addition, stomatal density has a strongly supported positive association with elevation, while wood density has associations with both temperature (positive) and rainfall (negative) variables. Estimated correlation coefficients for the BayesTraits analyses ranged from −0.513 to 0.627 in the 1 × 1 analyses, but only eight of 110 were individually very strongly supported. Nine additional correlations were strongly supported, 17 were weakly supported, and the remaining 76 lacked substantial support. The most strongly supported evolutionary correlations were between plant size (height) and variables related to elevation or temperature (mat, elevation, and *t*_min_), where taller plant are found in warmer areas (Figure 3, Table S5). Eight comparisons had a Bayes factor that exceeded the threshold of 9.7 to hold across all comparisons (Figure 3).

### 3.2 Models of evolution

Morphological and climatic‐niche traits appear to evolve in largely similar ways when analyses are based on the “best” tree and on trait or climate variable means for each species. All analyses of morphological traits for the 1 × 1 analysis (best tree and mean trait value) favored the OU model that indicates evolution around an optimum, or phylogenetic inertia in traits with limits on trait variance (Table 2, Table S6). Values for the alpha parameter are often quite high, although the OU model is still preferred over white noise (for which alpha is equal to infinity). Thus, the morphological traits of descendants are largely similar to those of their ancestors, and the range of morphological trait variation in the genus is somewhat constrained. Analyses of climatic‐niche traits also favored OU models for climate variables except for *t*_var_ (which followed a white noise model) when analyses are based on the “best” tree and on species' climate means (Table 2). Incorporating uncertainty (in the 1 × 100, 100 × 1, and 100 × 100 analyses) resulted in largely the same results for traits, although some replicates included support for BM or white noise models (particularly in canopy area, leafarea, and wood density; Figure S2, Table S6). For climate niche, incorporation of uncertainty in niche trait values resulted more often in white noise, especially with climatic‐niche uncertainty, which may be an artifact of the MaxEnt based resampling scheme (Figure S2).

**Table 2:** Comparisons of models of evolution for all traits and climate variables under Brownian motion (BM), Ornstein–Uhlenbeck (OU), and white noise (WN) in the 1 × 1 analyses

Traits						Climate variables					
Variable	Model	Log‐likelihood	AIC*c*	Akaike weight	Parameter estimates	Variable	Model	Log‐likelihood	AIC*c*	Akaike weight	Parameter estimates
height	BM	−83.163	170.544	0.007	σ^2^ = 2.793	mat	BM	13.477	−22.735	0.032	σ^2^ = 0.010
	**OU**	−77.151	160.746	0.984	α = 3.085, σ^2^ = 6.221		**OU**	17.994	−29.544	0.957	α = 2.580, σ^2^ = 0.206
	WN	−83.05	170.318	0.008	σ^2^ = 1.026244		WN	12.472	−20.727	0.012	σ^2^ = 0.038
canopy	BM	−110.606	225.43	0.006	σ^2^ = 7.196343	map	BM	−172.664	349.545	0	σ^2^ = 61.160
	**OU**	−104.539	215.523	0.81	α = 3.238, σ^2^ = 16.928		**OU**	−158.475	323.394	0.909	α = 4.986, σ^2^ = 151.688
	WN	−107.134	218.486	0.184	σ^2^ = 2.355		WN	−161.884	327.986	0.091	σ^2^ = 15.554
leafarea	BM	−95.876	195.971	0.456	σ^2^ = 4.330	elev	BM	−203.799	411.817	0.026	σ^2^ = 178.957
	**OU**	−94.587	195.619	0.543	α = 1.218, σ^2^ = 6.580		**OU**	−199.064	404.572	0.97	α = 2.714, σ^2^ = 379.325
	WN	−101.82	207.857	0.001	σ^2^ = 1.960		WN	−205.646	415.511	0.004	σ^2^ = 70.342
wood	BM	26.862	−49.483	0.249	σ^2^ = 0.050	pet	BM	−134.066	272.351	0	σ^2^ = 16.160
	**OU**	28.697	−50.904	0.507	α = 2.249, σ^2^ = 0.109		**OU**	−117.499	241.442	0.902	α = 12.101, σ^2^ = 83.520
	WN	26.844	−49.448	0.245	σ^2^ = 0.021		WN	−120.832	245.882	0.098	σ^2^ = 3.776
lma	BM	−3.058	10.334	0.353	σ^2^ = 0.176	rfl2 mm	BM	−61.022	126.263	0	σ^2^ = 1.3019
	**OU**	−1.341	9.126	0.646	α = 1.406, σ^2^ = 0.282		**OU**	−49.847	106.138	0.994	α = 3.933, σ^2^ = 2.934
	WN	−8.641	9.126	0.001	σ^2^ = 0.079		WN	−56.069	116.357	0.006	σ^2^ = 0.405
lwr	BM	−62.619	129.456	0.344	σ^2^ = 1.376	rflcv	BM	−36.441	77.1	0	σ^2^ = 0.558
	**OU**	−60.874	128.191	0.648	α = 1.742, σ^2^ = 2.446		**OU**	−27.077	60.598	0.771	α = 5.052, σ^2^ = 1.652
	WN	−66.404	137.025	0.008	σ^2^ = 0.578		WN	−29.406	63.03	0.229	σ^2^ = 0.161
fwc	BM	3.87	−3.514	0.161	σ^2^ = 0.118	summer	BM	−159.984	324.186	0.098	σ^2^ = 39.498
	**OU**	6.563	−6.665	0.778	α = 1.880, σ^2^ = 0.225		**OU**	−156.655	319.754	0.902	α = 1.627, σ^2^ = 64.111
	WN	2.896	−1.565	0.061	σ^2^ = 0.053		WN	−177.026	358.27	0	σ^2^ = 26.219
sd	BM	−27.574	59.367	0.004	σ^2^ = 0.411	*t*_max_	BM	20.967	−37.716	0.015	σ^2^ = 0.077
	**OU**	−21.004	48.453	0.995	α = 2.808, σ^2^ = 0.837		**OU**	26.269	−46.094	0.979	α = 2.792, σ^2^ = 0.163
	WN	−29.356	62.929	0.001	σ^2^ = 0.161		WN	20.032	−35.845	0.006	σ^2^ = 0.029
nmass	BM	5.774	−7.321	0.184	σ^2^ = 0.111	*t*_min_	BM	−141.101	286.421	0	σ^2^ = 20.597
	**OU**	8.317	−10.172	0.765	α = 1.891, σ^2^ = 0.212		**OU**	−131.398	269.24	0.523	α = 5.874, σ^2^ = 68.848
	WN	4.481	−4.735	0.051	σ^2^ = 0.050		WN	−132.602	269.24	0.477	σ^2^ = 5.667
δ13C	BM	−84.165	172.556	0.203	σ^2^ = 2.746	*t*_var_	BM	10.55	−16.883	0	σ^2^ = 0.110
	**OU**	−81.685	169.831	0.792	α = 1.50, σ^2^ = 4.624		OU	30.181	−53.917	0.266	α = 48.962, σ^2^ = 2.026
	WN	−87.893	180.013	0.005	σ^2^ = 1.351		**WN**	30.082	−55.946	0.734	σ^2^ = 0.021
cnratio	BM	6	−7.773	0.243	σ^2^ = 0.110						
	**OU**	8.221	−9.981	0.734	α = 1.657, σ^2^ = 0.197						
	WN	3.599	−2.972	0.022	σ^2^ = 0.051						

Models were compared using AICc as well as Akaike weights. The “best” model for each trait/climate is bolded.

### 3.3 Correlated evolution along axes of variation

**Figure 4:**
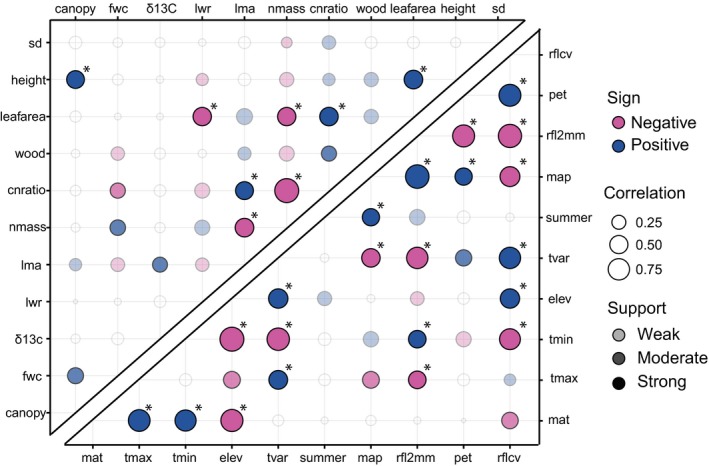
Trait–trait and climate–climate integration. Sign, strength, and significance of correlations among traits (upper left panel) and among climate variables (lowe right panel) for evolutionary correlations based on BayesTraits. Correlations are either positive (blue) or negative (magenta), vary in strength (size of circle), and have different levels of support indicated by transparency of circle color (weak support: logBF > 2, *p* < .10, most transparent; moderate support: logBF > 5, *p* < .05, medium transparency; strong support: logBF > 10, *p* < .01, darkest circles). Correlations not supported at any level have no fill (completely transparent). Asterisks indicate correlations that meet the criterion threshold correcting for multiple comparisons

BayesTraits analyses reveal a wide range of pairwise correlations among morphological traits and among climatic variables (Figure 4). We identified two major axes of morphological traits with strong patterns of covariation based on BayesTraits correlations: (1) general size (leafarea, height, canopy area) and (2) leaf composition (leafarea, cnratio, lma, δ13C, lwr, nmass). Leaf area is a component of both of these, while wood density also has weak to moderate connections with both axes. Stomatal density (sd) appears to evolve somewhat independently of the other traits (Figure 4). Among morphological traits, estimated correlations ranged from −0.994 to 0.532 in the 1 × 1 analyses. Eight of the 55 correlations had very strong support (logBF > 10), five had strong support (10 > logBF > 5), and 16 had weak support (5 > logBF > 2). The remaining 26 had very weak support (logBF < 2; Figure 4, Table S5). Eight of these met the Bayes factor threshold of 9.8 adjusting for multiple comparisons (Figure 4).

We identified three major suites of climatic variables with strong patterns of covariation: (1) temperature (mat, *t*_min_, and *t*_max_), (2) rainfall (summer_rain, map, rfl2 mm), and (3) a group combining temperature, rainfall, and variability (pet, rflcv, elev, *t*_var_; Figure 4). There is also an association between the temperature and rainfall axes of variation. Estimated Bayes Traits pairwise correlations for climate variables ranged from −0.905 to 0.865 in the 1 × 1 analyses. Twenty‐one of the 45 pairwise correlations were very strongly supported, four were strongly supported, six were weakly supported, and 14 lacked support (Figure 4, Table S5). The strongest correlations were those between elevation and *t*_min_ (corr = −0.905, logB = 94.5) and map and rfl2 mm (corr = 0.865, logBF = 78.8). Twenty‐one of these met the Bayes factor threshold of 9.6 adjusting for multiple comparisons (Figure 4).

**Figure 5:**
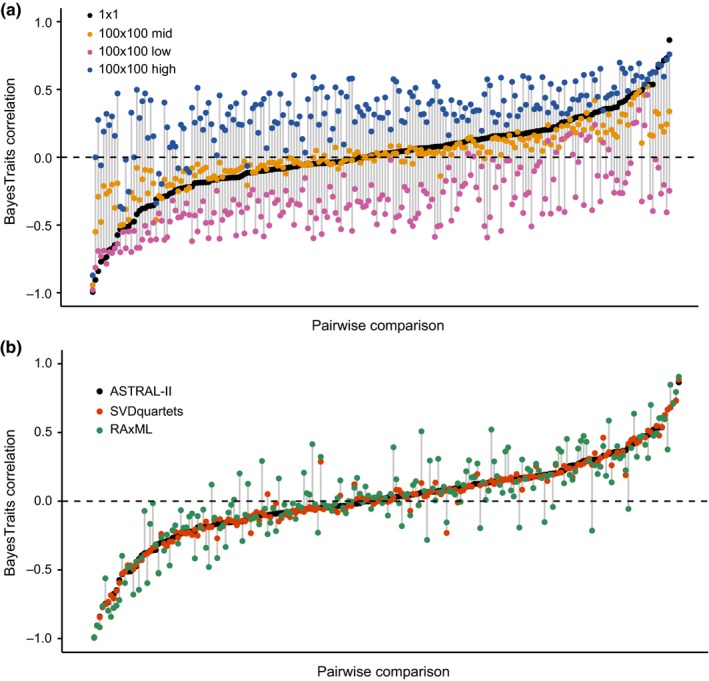
Uncertainty in BayesTraits analyses. (a) Comparisons of the correlation coefficients from the 1 × 1 (mean observation x best tree, black) with the median (gold), low (2.5%, magenta), and high (97.5%, blue) values from the 100 × 100 analyses for all 210 comparisons. Pairwise comparisons are arbitrarily sorted by the 1 × 1 correlation value. (b) Comparisons of 1 × 1 values from the ASTRAL‐II (black), SVDquartets (orange), and RAxML (green) best species trees

Incorporating trait uncertainty across all 210 pairwise BayesTraits associations (100 × 100 datasets) resulted in qualitatively similar outcomes (Figure 5a). The median values of the correlations for the 100 × 100 dataset were similar to the values from 1 × 1 analyses, although they tended to be less extreme. Notably, the upper and lower (97.5th and 2.5th percentiles) indicate that point estimates are very imprecise. In contrast, using other species trees has a minor influence on point estimates using other species trees, and the interval of the estimates is fairly narrow, indicating that phylogenetic uncertainty is not contributing heavily to differences in these estimates (Figure 5b).

## 4 DISCUSSION

### 4.1 Correlated trait–climate evolution indicative of adaptive radiation

Our results provide several examples where morphological traits are correlated with climatic‐niche variables when taking into account evolutionary history. Although these data are collected in the field and patterns could be due to phenotypic plasticity, we find similar trait–climate relationships in this and previous studies within a subset of the region (Mitchell et al., 2015) and in experimental common‐garden studies (Carlson et al., 2011; Prunier et al., 2012). The overall patterns found here involve the same two dimensions of variation in plant traits found in the global analysis by Díaz et al. (2016): plant size and leaf economics. Here, we find a positive association between plant and leaf size and temperature, and a negative association between leaf construction cost (e.g., LMA) and rainfall amount.

The finding of correlated trait–climate evolution along two main axes is consistent with previous work in *Protea,* suggesting that trait–climate associations are adaptive. For example, Carlson et al. (2011) showed in the white proteas (a strongly supported clade of six species: Mitchell et al., 2017) that trait‐performance differences in two experimental gardens are consistent with patterns expected from trait–climate associations in wild populations and that in‐garden survival is associated with trait differences in the predicted way. That work provided direct evidence that many trait–climate associations are adaptive. Similarly, selection gradient analyses using an estimate of lifetime seed production as a proxy for fitness demonstrated differential selection in two wild populations consistent with predictions of trait–climate associations in *P. repens* (Carlson et al., 2015). Although the results here do not provide direct evidence for adaptation, they suggest that adaptive differences identified within species and among closely related species may be extrapolated across the entire genus.

Plants commonly tend to be taller and have larger leaves with dense wood in warm areas and to be shorter with smaller leaves and less dense wood at high elevations (see Kdimer, 1999), reflecting both hydraulic and allometric constraints to woody plant height (Givnish, Wong, Stuart‐Williams, Holloway‐Phillips, & Farquhar, 2014). In the CFR, this association may additionally be confounded with soil depth, where lowland areas generally have deeper soil than high elevation sites (Campbell and Werger 1988). Our associations between plant size and temperature are consistent with the finding that mean annual temperature is generally a better predictor of plant traits than mean annual precipitation (Moles et al., 2014). In contrast, Moles et al., 2009 found that precipitation during the wettest month was most strongly associated with plant height, while Thuiller, Lavorel, Midgley, Lavergne, and Rebelo (2004) found no association between climate and plant height and found that leaf size was correlated with precipitation, not temperature, in the related genus *Leucadendron*. Yates, Anthony Verboom, Rebelo, and Cramer (2010) concluded that leaf size in Proteaceae may reflect the ability to dissipate heat during warm periods and quickly transpire during wet periods, although our patterns of leaf size and temperature are opposite to their findings.

The other broad pattern that we detected is that “slow” trait values on the leaf economics spectrum are associated with lower levels of rainfall. Plants in areas with low rainfall invest more in their physical construction, with higher values of LMA and wood density, higher C:N ratios, lower nmass, and lower leaf water contents. These results are consistent with the hypothesis that higher values of LMA are associated with optimizing plant growth in drier or nutrient‐poor areas (Givnish, 1979; Reich et al. 2003, Wright et al., 2005), but they may be inconsistent with previous results, suggesting that temperature is more strongly associated with higher carbon investment in leaf construction (Moles et al., 2014). Lamont, Groom, and Cowling (2002) tested these associations more explicitly using Proteaceae species in both South Africa and Australia along precipitation gradients. They found that LMA was inversely correlated with rainfall and that this relationship is perhaps more important than soil nutrient status.

Wood density is a component of both suites of traits, and its positive association with temperature and negative association with precipitation are consistent with global patterns (Swenson & Enquist, 2007) and may reflect an adaptation to decrease the chance of xylem cavitation during periods of drought stress and the lack of water availability associated with hotter and drier downslope regions in the CFR (Hacke, Sperry, Pockman, Davis, & McCulloh, 2001).

Similar associations between the evolution of morphological traits and shifts in climatic‐niche have also been seen in *Pelargonium* in the CFR (Jones et al., 2013), but the trait–climate associations found in *Protea* and *Pelargonium* can differ (Mitchell et al., 2015). For example, larger plants are associated with more drought in *Pelargonium*, while the opposite is true in *Protea*. These differences may be due to the occupation of unique microhabitats or differing growth forms and life history strategies. Similarly, the lack of detectable associations between leaf mass per area and temperature variables is not at odds with the weak negative association in evergreen plants on the global scale, while the negative relationship with summer rain here is consistent with the global relationships across evergreens (Wright et al., 2005). Precipitation may be a more important driver than temperature for LMA in evergreen plants in the CFR, where rainfall amount is more variable and results in periods of drought stress (as opposed to tropical regions).

### 4.2 Models of trait and climatic‐niche evolution

Morphological traits are evolutionarily conserved in *Protea*, which may be the result of phylogenetic niche conservatism. An Ornstein–Uhlenbeck model consistently provides the best fit to data from morphological traits, indicating that the morphological traits of descendants not only resemble those of their ancestors but also that the range of variation within the genus is bounded (Cooper, Jetz, & Freckleton, 2010). Similarly, OU models provide the best fit to the data for climatic‐niche traits (with the exception of *t*_var_), suggesting that there is also some degree of phylogenetic niche conservatism around optima in niches. Comparisons with both Brownian motion and white noise models lend greater support for phylogenetic niche conservatism, despite high values of the alpha parameter for several traits. The finding of phylogenetic niche conservatism is not unexpected, as Skeels and Cardillo (2017) found evidence for multiple‐optima OU models demonstrating phylogenetic niche conservatism in *Protea,* with differences between hotspot and nonhotspot clades.

### 4.3 Integrated trait evolution

Analysis of both contemporary and evolutionary trait associations provides evidence for covariation of morphological traits and climate variables in *Protea*. Individual morphological traits and climatic‐niche variables are used to indicate significant aspects of the “whole organism” and “*n*‐dimensional hypervolume” climatic‐niche variation (Hutchinson, 1957; Reich, 2014; Reich et al., 2003). Correlations among morphological traits are one way of measuring the degree to which phenotypes are integrated, whether as a result of shared function, development, or genetics (Pigliucci, 2003). Patterns of integration may be especially interesting when analyzing traits across organ systems or those involved with different physiological processes in plants. For instance, hydraulic capacity in plant stems can be linked to both hydraulic and photosynthetic rates in leaves (Brodribb, Holbrook, & Gutiérrez, 2002; Sack, Cowan, Jaikumar, & Holbrook, 2003). Similarities or differences between contemporary and evolutionary associations between morphological traits may provide clues to the causes of phenotypic integration. For example, the strong negative associations between height and canopy area in both sets of analyses could reflect fundamental biophysical constraints. In contrast, the negative association between lma and δ ^13^C (sclerophyllous leaves with higher lma have more negative δ^13^C values (less water use efficient)) may reflect a functional association between strategies enhancing resource conservation and those enhancing water use efficiency.

Some patterns of trait integration in *Protea* are consistent with those previously seen in global datasets. In plants, several different syndromes of integrated traits have been proposed including Chapin et al.'s “stress‐resistance syndrome” (Chapin, Autumn, & Pugnaire, 1993), Westoby's LHS strategy (1998, and the many variations of the worldwide leaf (or whole plant) economics spectrum (LES; Reich, 2014; Wright et al., 2004). Each of these syndromes involves suites of correlated traits, combined with trade‐offs among them. In *Protea*, for example, we find a positive evolutionary association between cnratio and lma and a negative association between nmass and lma, consistent with worldwide patterns in the LES. Similarly, we find (1) a positive evolutionary association between leafarea and plant height and then between plant height and canopy area, and (2) a positive association between leafarea and cnratio and a corresponding negative association between leafarea and nmass, suggesting that there are integrated traits related to overall investment and plant size (Figure 4a). At the evolutionary scale, wood density is weakly or moderately linked to attributes of both axes (plant size and leaf investment), while stomatal density evolves largely independently. Wood density may be bridging the gap between the suites of trait, as it can be tied to plant size and physical support associated with temperature, and the need for cavitation resistance associated with low rainfall.

Not surprisingly, several climatic‐niche variables also covary. As the climatic‐niches descendants occupy are, at best, weakly correlated with those of their progenitors, these associations probably reflect intrinsic physical climate correlations rather than correlated niche evolution. For example, the positive network of mat, *t*_max_, and *t*_min_, and their negative associations with elevation are expected due to adiabatic cooling and would likely be detected in any random sample of geographic locations. In contrast, the associations between seasonality, precipitation, and temperature reflect niche hypervolumes characteristic of the CFR and perhaps of other Mediterranean climate regions around the world characterized by cool, wet winters and hot, dry summers.

### 4.4 Incorporating uncertainty in comparative analyses

Two sources of uncertainty should be recognized in any comparative analysis: (1) uncertainty about trait values for the species that arises because of trait variation within species and (2) uncertainty about species relationships that arises because phylogenetic relationships are imperfectly estimated. In our case, incorporating these sources of uncertainty did not qualitatively affect our results in the BayesTraits analyses. The median values incorporating both intraspecific trait variation and phylogenetic uncertainty (i.e., the 100 × 100 analyses) were very similar to those from the 1 × 1 analyses based on the species mean value and the “best” phylogenetic tree. Nonetheless, the range of values generated in the 100 × 100 analyses indicates that results using any single point estimate, such as a mean, must be interpreted with considerable caution. This is particularly evident in the analysis of evolutionary models for climatic‐niche traits, where an Ornstein–Uhlenbeck model was supported in the 1 × 1 analysis for all but *t*_var_, while white noise models were often favored in the 100 × 100 analyses, although this may be due to the nonnormal sampling from the MaxEnt output.

## 5 CONCLUSIONS

The correlated evolution of traits and climatic‐niches in *Protea*, combined with previous experimental work in the group, suggests that broadly adaptive processes played an important role in divergence of many morphological traits during this radiation. Moreover, our results provide evidence for two different suites of trait–climate associations, possibly reflecting a combination of fundamental biophysical or functional relationships among traits and intrinsic physical properties of climate variables. In particular, we identified that the same two dimensions of plant variation found at the global scale are associated with different aspects of the climate, namely: (1) a positive association between plant size and temperature, and (2) a “faster” leaf investment strategy with higher amounts of rainfall. These are supported by consistent findings between evolutionary and contemporary associations. Phenotypic traits are relatively conserved, which may be accounted for by climatic‐niche conservatism. Overall, we find substantial evidence for broadly adaptive coevolution among traits and climate even when incorporating both uncertainty in phylogenetic relationships and to within‐species variation in trait values. Together with past work demonstrating that these traits are adaptive and heritable, we find evidence that the radiation of *Protea* is indeed adaptive. Future work on trait and physiological differentiation in closely related co‐occurring species of *Protea* may provide more robust evidence for the mechanisms underlying these phenotype‐climate associations.

## CONFLICT OF INTEREST

None declared.

## AUTHOR CONTRIBUTIONS

N. Mitchell performed a large portion of the trait data collection, conceived of and carried out all analyses, and wrote the majority of the text. J.E. Carlson oversaw and carried out collection of the trait data and gave crucial feedback on the manuscript. K.E. Holsinger obtained grant funding, oversaw the project, contributed to edits, and helped with structuring the manuscript.
