# Supplementary material for: Correlated evolution between climate and suites of traits along a fast–slow continuum in the radiation of Protea
Source: Ecol Evol. 2018 Jan 12;8(3):1853–66. doi: 10.1002/ece3.3773 (PMC5792567; doi:10.1002/ece3.3773)
Supplement: Supplementary file 1 [file ECE3-8-1853-s001.pdf]

log(height)

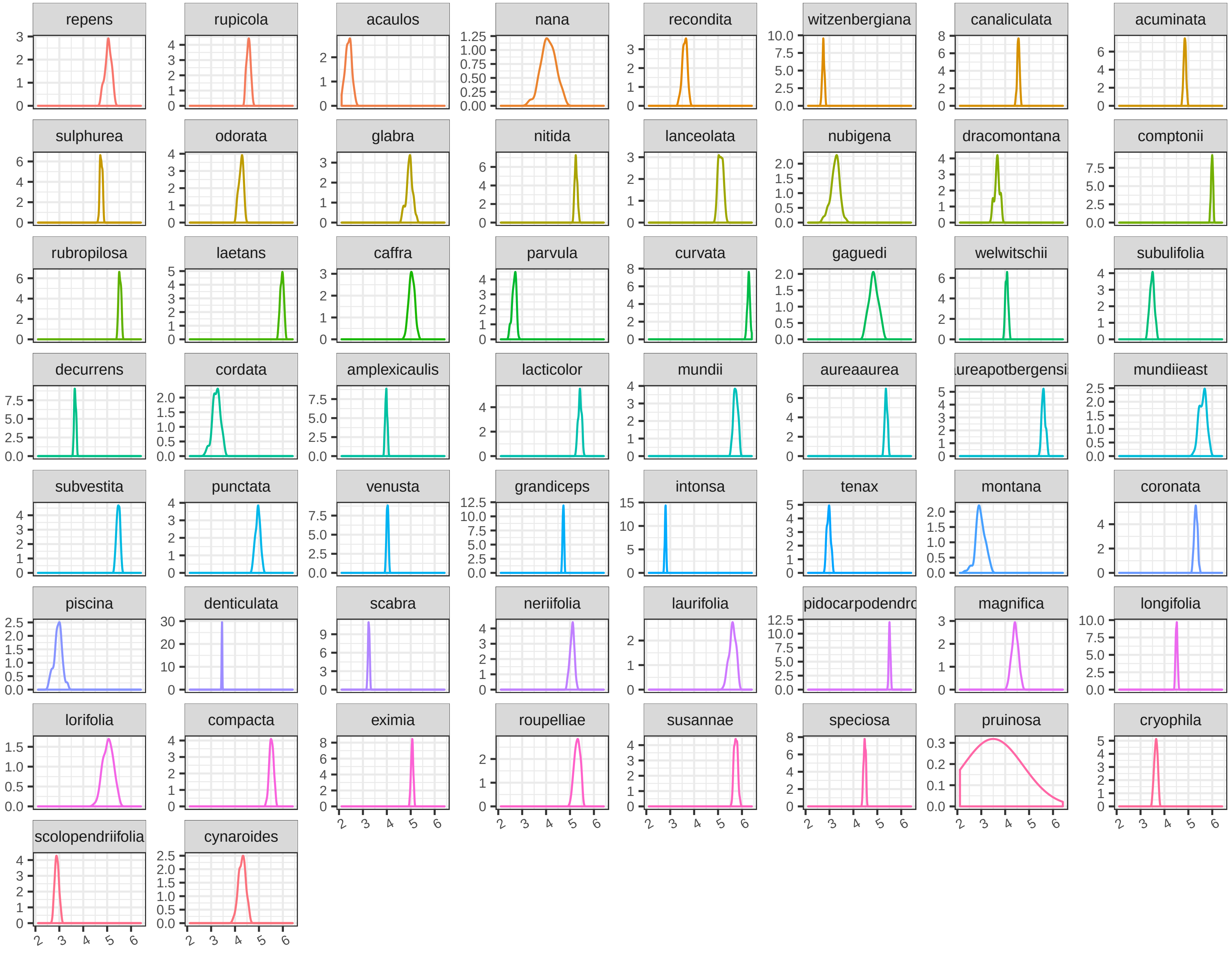

repens
rupicola
acaulos
nana
recondita
witzenbergiana
canaliculata
acuminata
sulphurea
odorata
glabra
nitida
lanceolata
nubigena
dracomontana
comptonii
rubropilosa
laetans
caffra
parvula
curvata
gaguedi
welwitschii
subulifolia
decurrens
cordata
amplexicaulis
lacticolor
mundii
aureaaurea
ureapotbergensi
mundiieast
subvestita
punctata
venusta
grandiceps
intonsa
tenax
montana
coronata
piscina
denticulata
scabra
neriifolia
laurifolia
pidocarpodendro
magnifica
longifolia
lorifolia
compacta
eximia
roupelliae
susannae
speciosa
pruinosa
cryophila
scolopendriifolia
cynaroides
2 3 4 5 6

log(canopy)

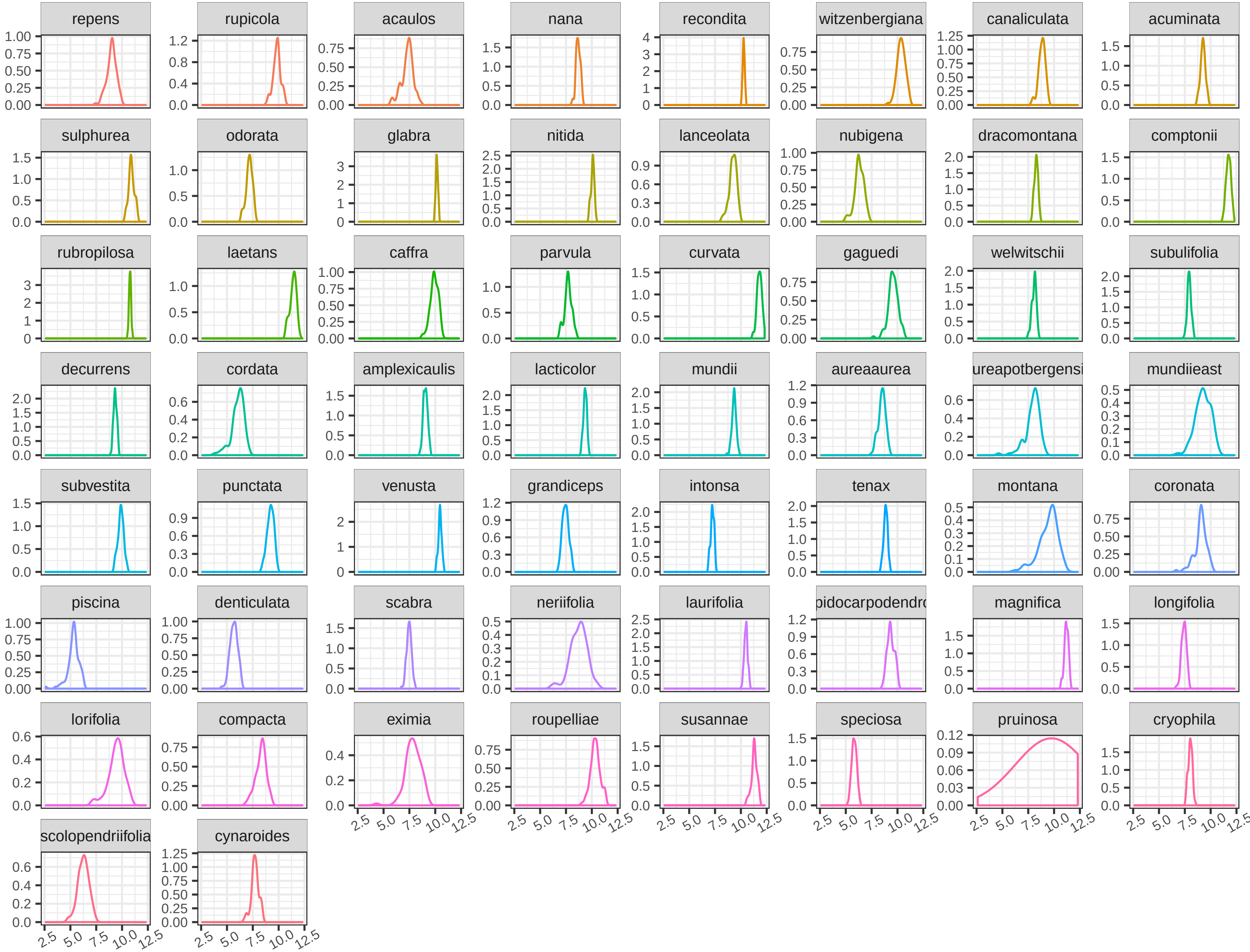

repens
rupicola
acaulos
nana
recondita
witzenbergiana
canaliculata
acuminata
sulphurea
odorata
glabra
nitida
lanceolata
nubigena
dracomontana
comptonii
rubropilosa
laetans
caffra
parvula
curvata
gaguedi
welwitschii
subulifolia
decurrens
cordata
amplexicaulis
lacticolor
mundii
aureaaurea
ureapotbergensi
mundiieast
subvestita
punctata
venusta
grandiceps
intonsa
tenax
montana
coronata
piscina
denticulata
scabra
neriifolia
laurifolia
pidocarpodendr
magnifica
longifolia
lorifolia
compacta
eximia
roupelliae
susannae
speciosa
pruinosa
cryophila
scolopendriifolia
cynaroides
2.5
5.0
7.5
10.0
12.5

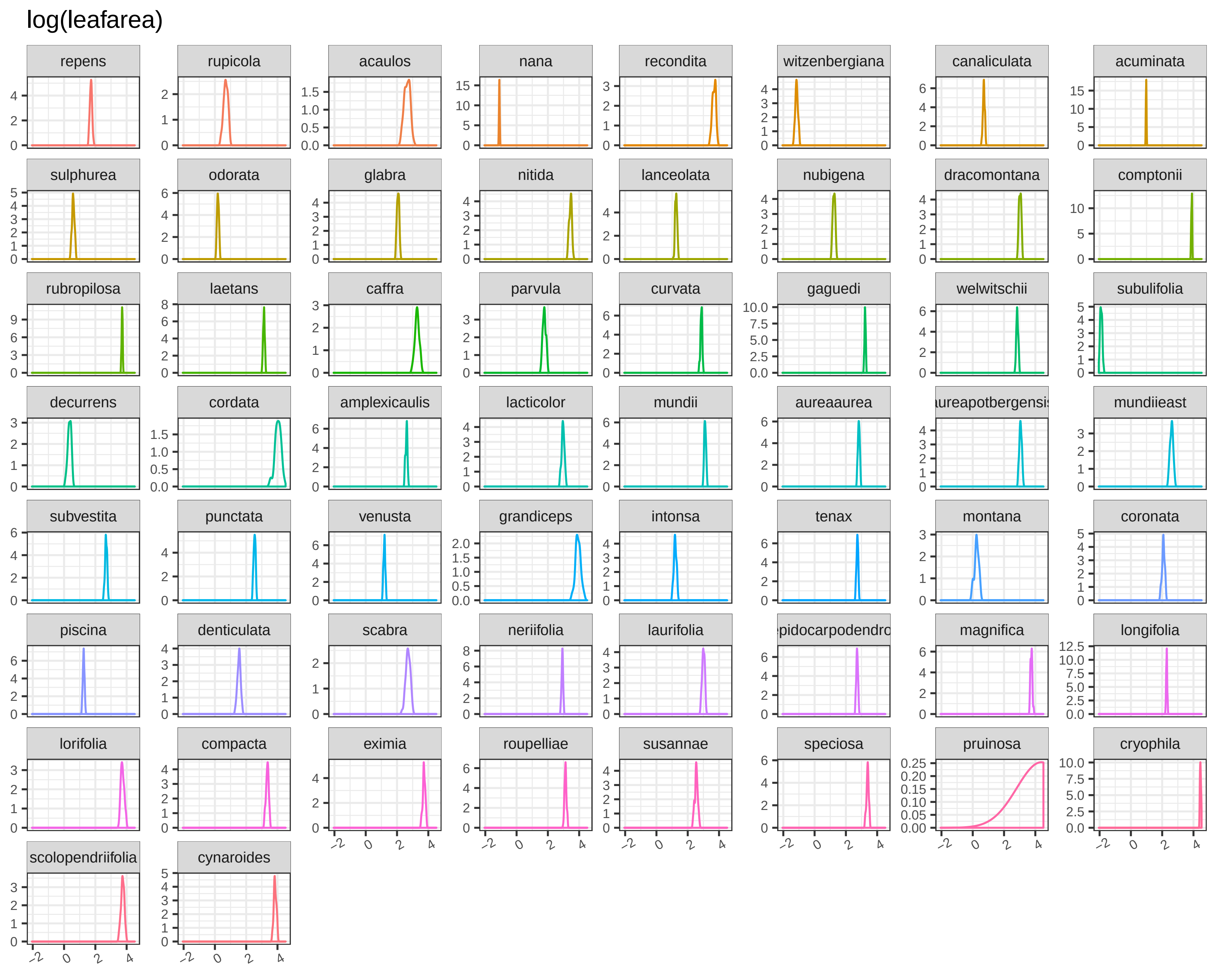

log(leafarea)
repens
rupicola
acaulos
nana
recondita
witzenbergiana
canaliculata
acuminata
sulphurea
odorata
glabra
nitida
lanceolata
nubigena
dracomontana
comptonii
rubropilosa
laetans
caffra
parvula
curvata
gaguedi
welwitschii
subulifolia
decurrens
cordata
amplexicaulis
lacticolor
mundii
aureaaurea
mundiieast
subvestita
punctata
venusta
grandiceps
intonsa
tenax
montana
coronata
piscina
denticulata
scabra
neriifolia
laurifolia
magnifica
longifolia
lorifolia
compacta
eximia
roupelliae
susannae
speciosa
pruinosa
cryophila
scolopendriifolia
cynaroides

log(wood)

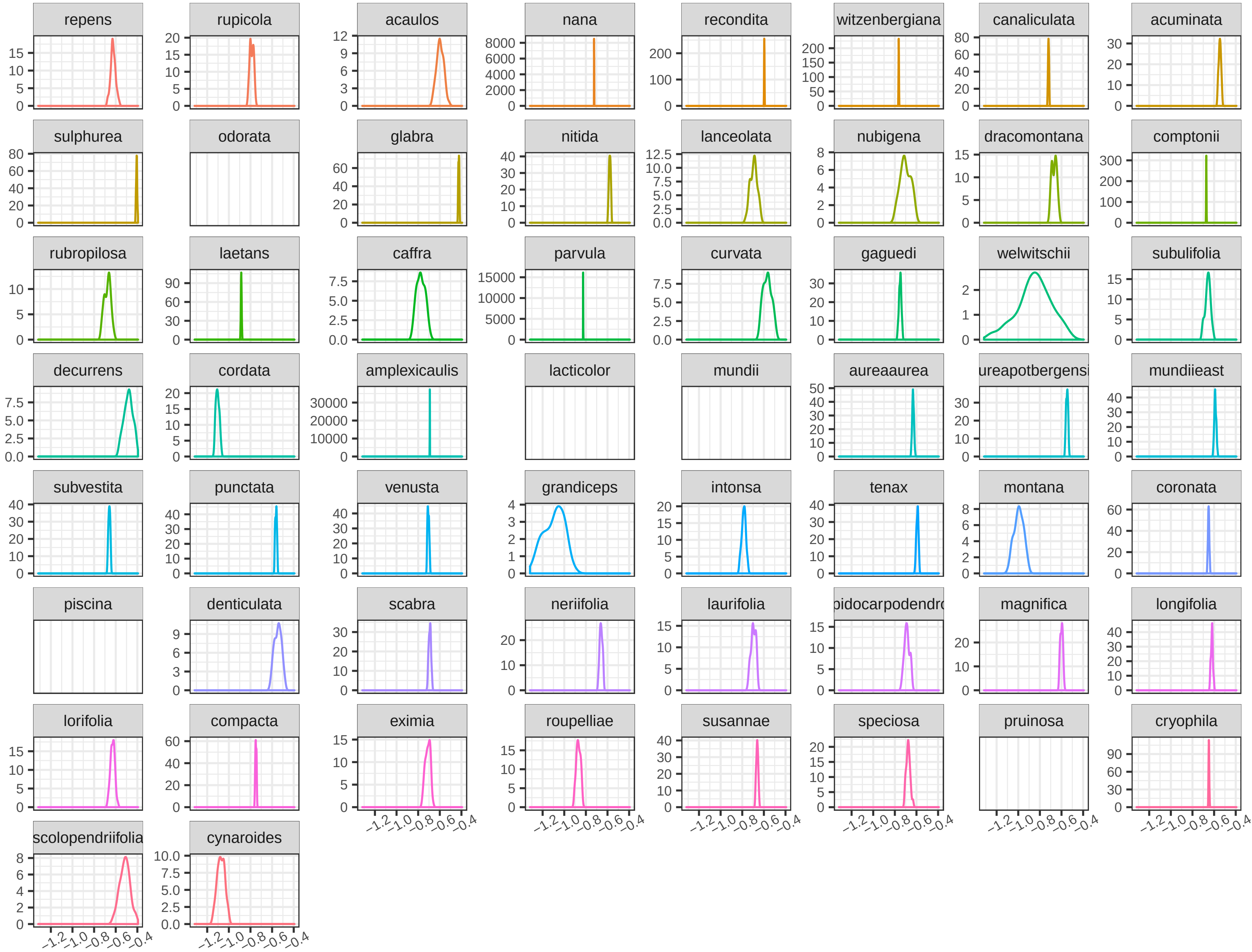
repens
rupicola
acaulos
nana
recondita
witzenbergiana
canaliculata
acuminata
sulphurea
odorata
glabra
nitida
lanceolata
nubigena
dracomontana
comptonii
rubropilosa
laetans
caffra
parvula
curvata
gaguedi
welwitschii
subulifolia
decurrens
cordata
amplexicaulis
lacticolor
mundii
aureaaurea
mundiieast
subvestita
punctata
venusta
grandiceps
intonsa
tenax
montana
coronata
piscina
denticulata
scabra
neriifolia
laurifolia
magnifica
longifolia
lorifolia
compacta
eximia
roupelliae
susannae
speciosa
pruinosa
cryophila
scolopendriifolia
cynaroides
−1.2
−1.0
−0.8
−0.6
−0.4

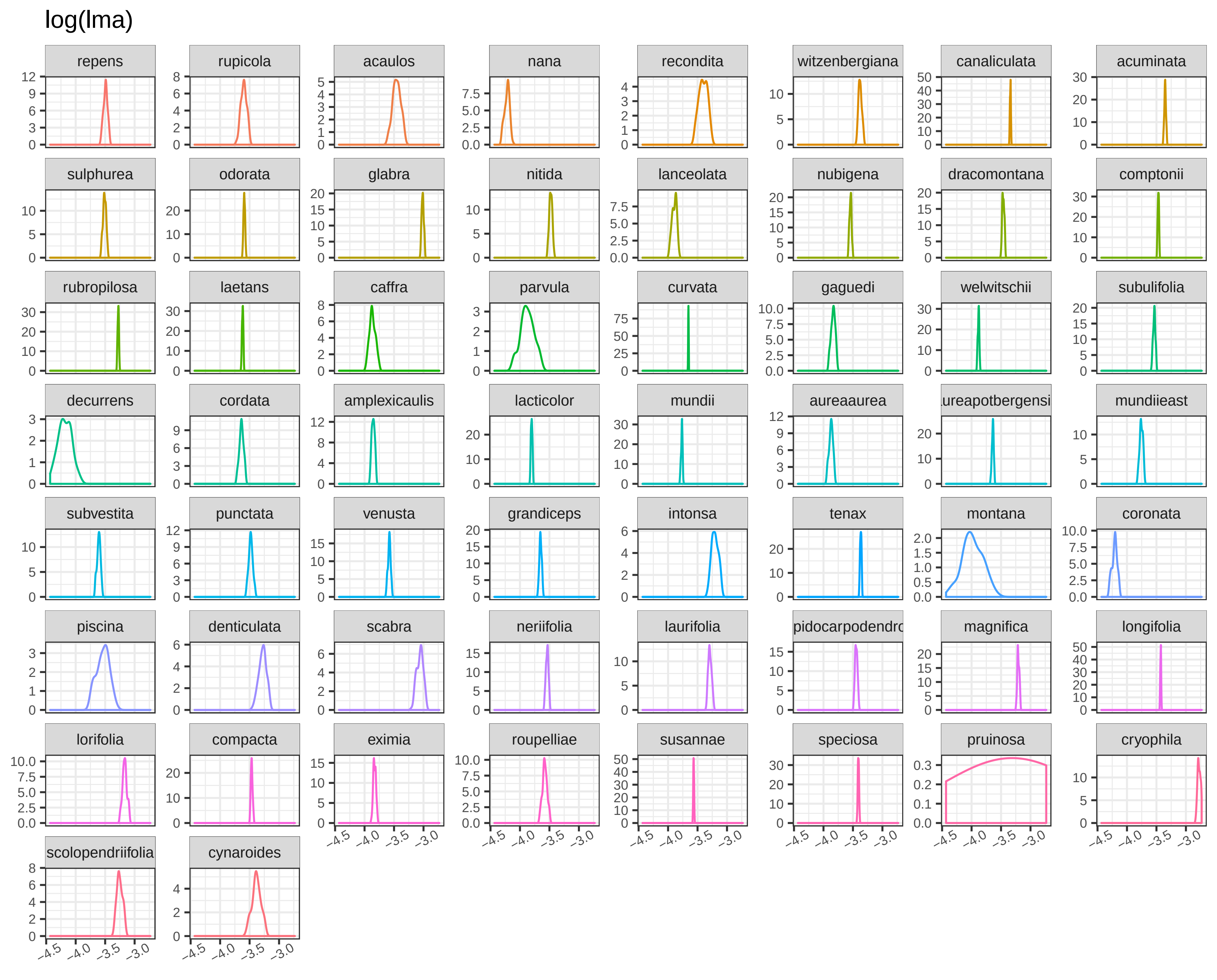
log(lma)
repens
rupicola
acaulos
nana
recondita
witzenbergiana
canaliculata
acuminata
sulphurea
odorata
glabra
nitida
lanceolata
nubigena
dracomontana
comptonii
rubropilosa
laetans
caffra
parvula
curvata
gaguedi
welwitschii
subulifolia
decurrens
cordata
amplexicaulis
lacticolor
mundii
aureaaurea
ureapotbergensi
mundiieast
subvestita
punctata
venusta
grandiceps
intonsa
tenax
montana
coronata
piscina
denticulata
scabra
neriifolia
laurifolia
pidocarpodendro
magnifica
longifolia
lorifolia
compacta
eximia
roupelliae
susannae
speciosa
pruinosa
cryophila
scolopendriifolia
cynaroides
-4.5
-4.0
-3.5
-3.0

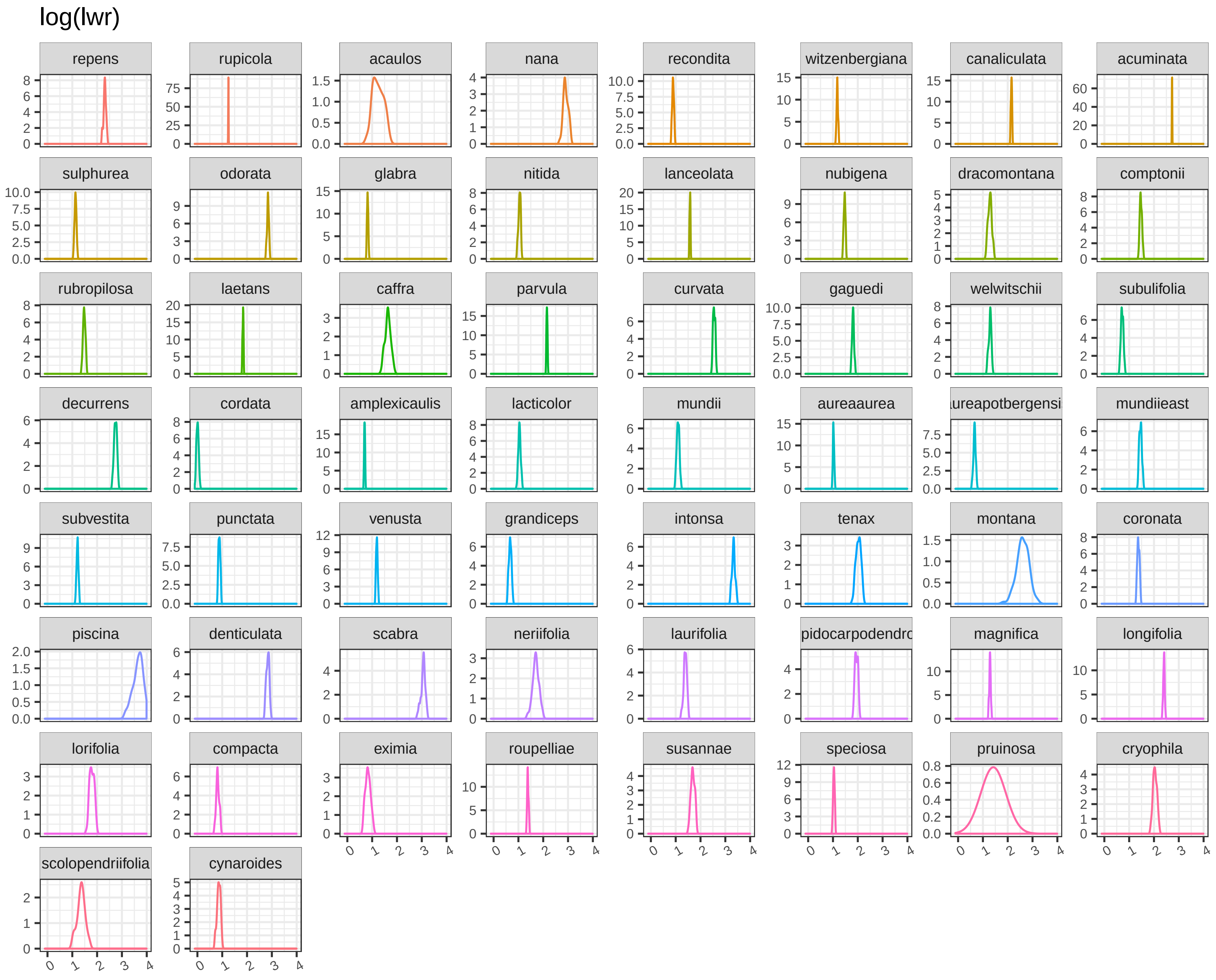

log(lwr)
repens
rupicola
acaulos
nana
recondita
witzenbergiana
canaliculata
acuminata
sulphurea
odorata
glabra
nitida
lanceolata
nubigena
dracomontana
comptonii
rubropilosa
laetans
caffra
parvula
curvata
gaguedi
welwitschii
subulifolia
decurrens
cordata
amplexicaulis
lacticolor
mundii
aureaaurea
ureapotbergensi
mundiieast
subvestita
punctata
venusta
grandiceps
intonsa
tenax
montana
coronata
piscina
denticulata
scabra
neriifolia
laurifolia
pidocarpodendro
magnifica
longifolia
lorifolia
compacta
eximia
roupelliae
susannae
speciosa
pruinosa
cryophila
scolopendriifolia
cynaroides

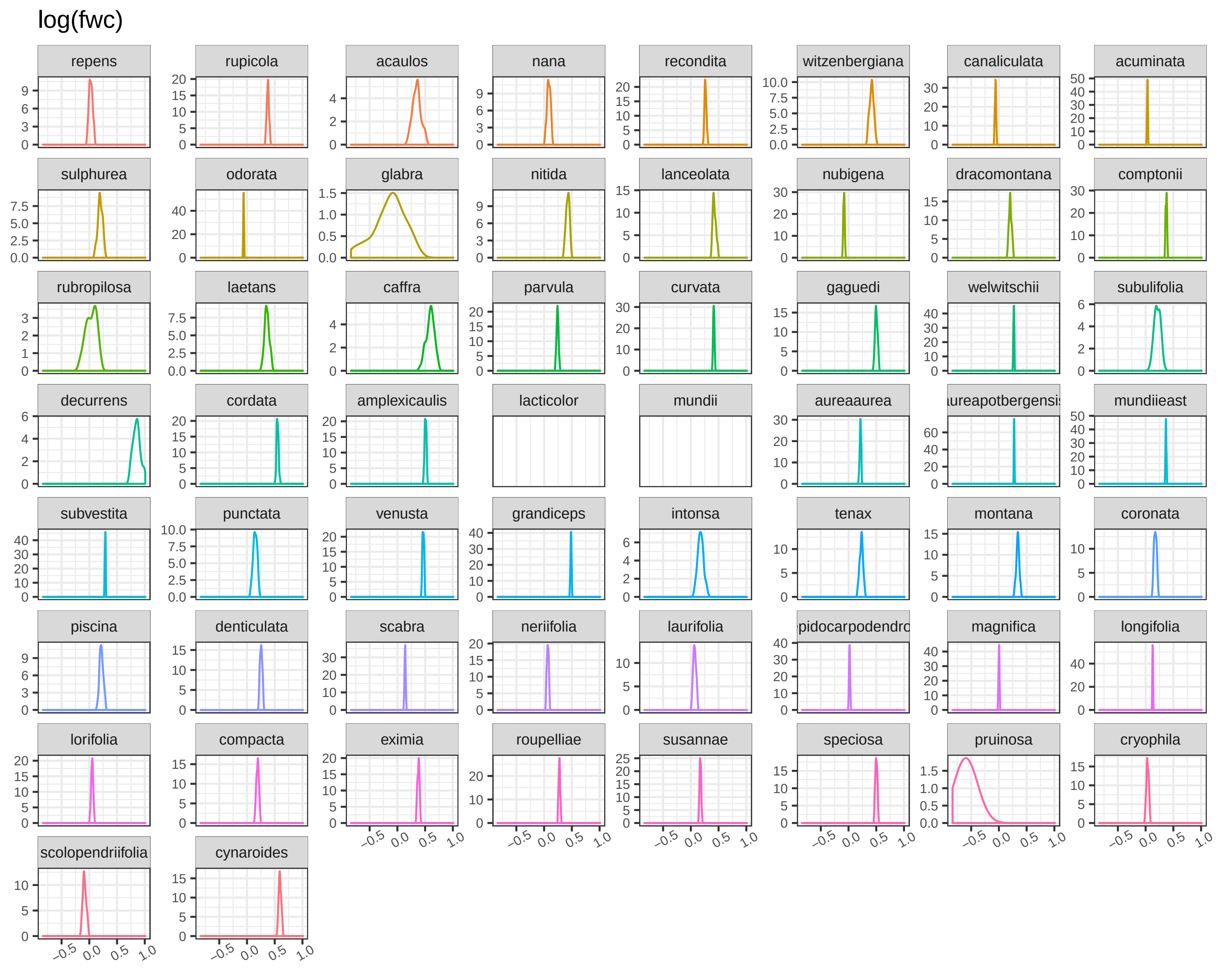

log(fwc)
repens
rupicola
acaulos
nana
recondita
witzenbergiana
canaliculata
acuminata
sulphurea
odorata
glabra
nitida
lanceolata
nubigena
dracomontana
comptonii
rubropilosa
laetans
caffra
parvula
curvata
gaguedi
welwitschii
subulifolia
decurrens
cordata
amplexicaulis
lacticolor
mundii
aureaaurea
ureapotbergensis
mundiieast
subvestita
punctata
venusta
grandiceps
intonsa
tenax
montana
coronata
piscina
denticulata
scabra
neriifolia
laurifolia
pidocarpodendro
magnifica
longifolia
lorifolia
compacta
eximia
roupelliae
susannae
speciosa
pruinosa
cryophila
scolopendriifolia
cynaroides
−0.5
0.0
0.5
1.0

log(sd)

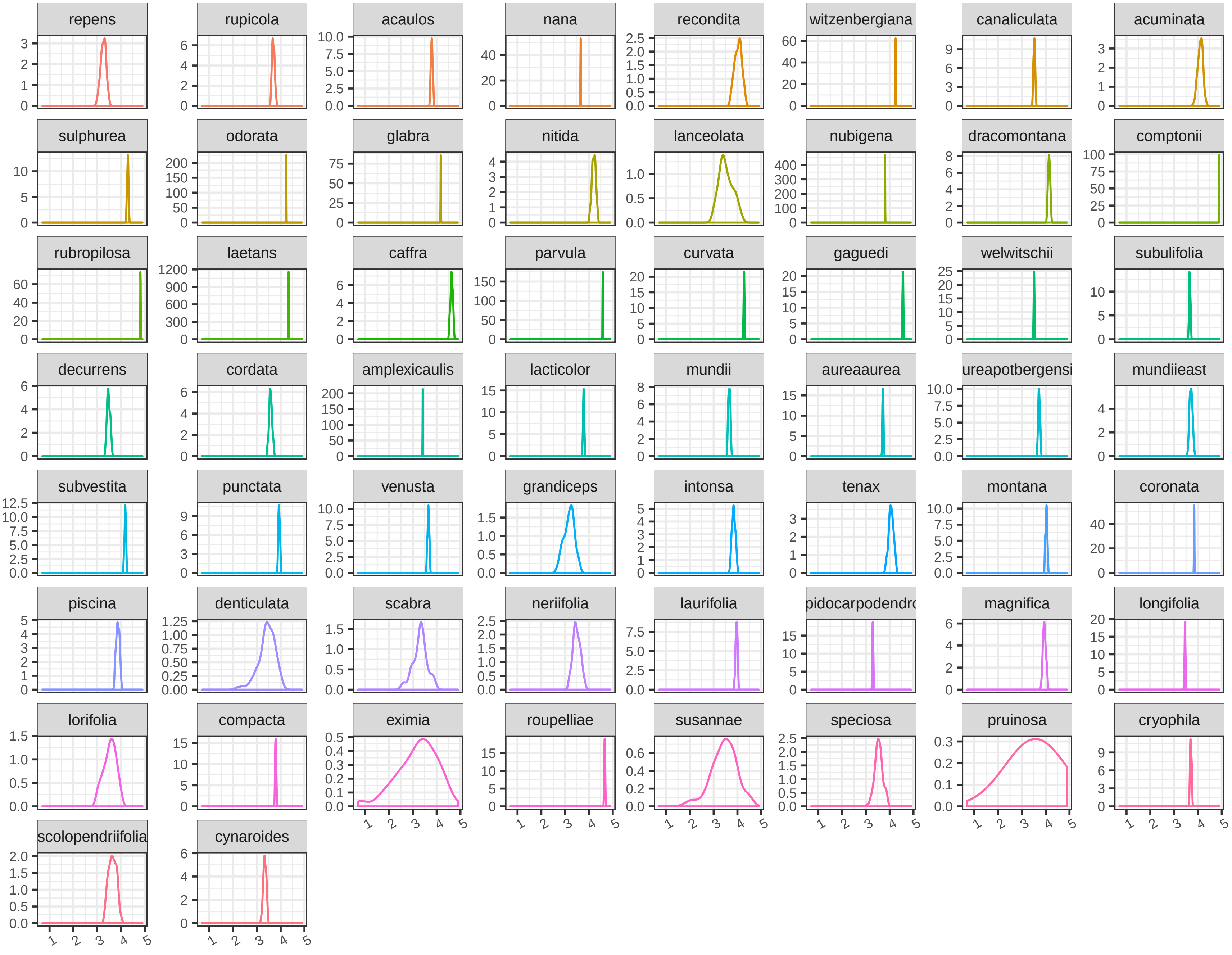

repens
rupicola
acaulos
nana
recondita
witzenbergiana
canaliculata
acuminata
sulphurea
odorata
glabra
nitida
lanceolata
nubigena
dracomontana
comptonii
rubropilosa
laetans
caffra
parvula
curvata
gaguedi
welwitschii
subulifolia
decurrens
cordata
amplexicaulis
lacticolor
mundii
aureaaurea
ureapotbergensi
mundiieast
subvestita
punctata
venusta
grandiceps
intonsa
tenax
montana
coronata
piscina
denticulata
scabra
neriifolia
laurifolia
pidocarpodendro
magnifica
longifolia
lorifolia
compacta
eximia
roupelliae
susannae
speciosa
pruinosa
cryophila
scolopendriifolia
cynaroides

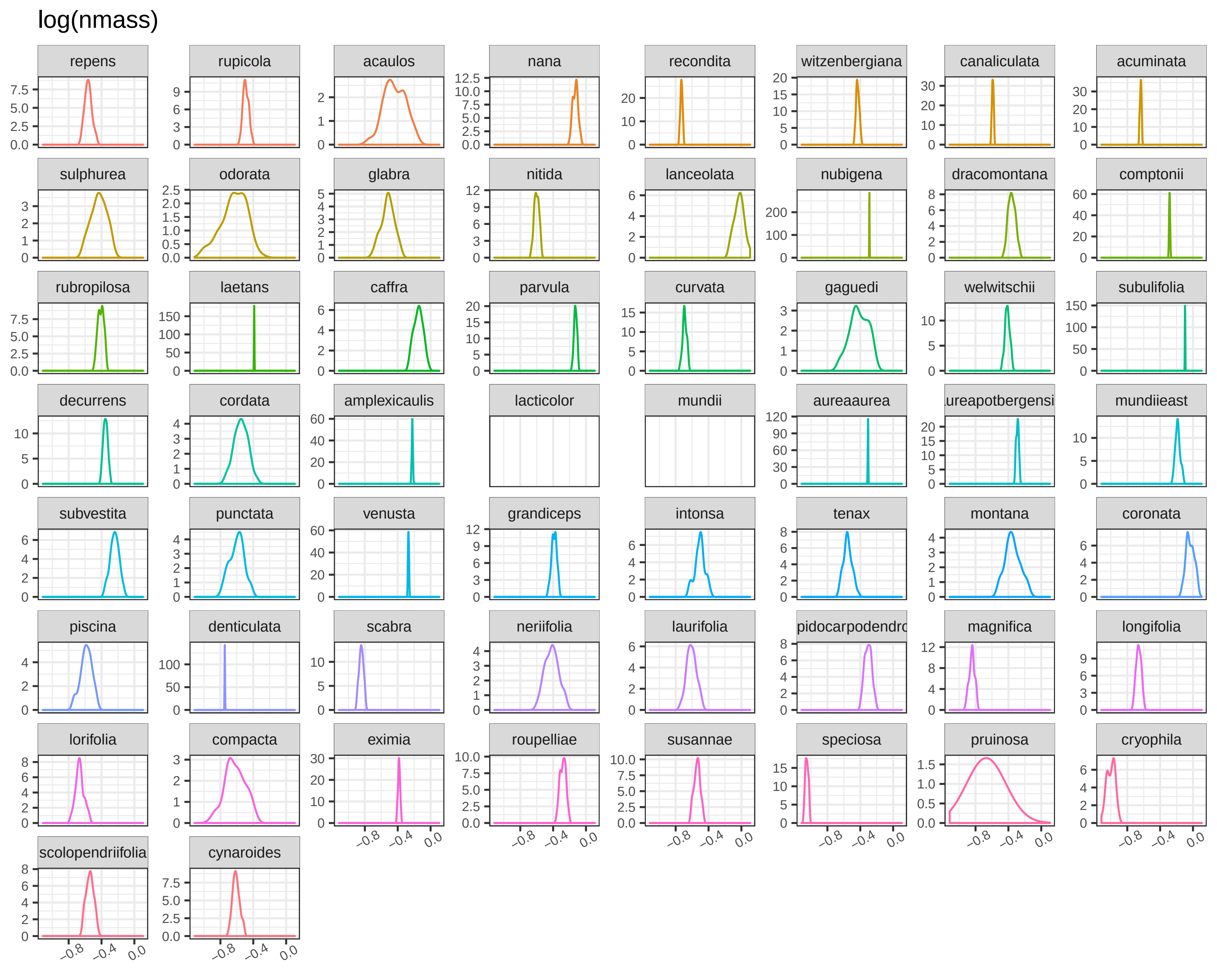

log(nmass)
repens
rupicola
acaulos
nana
recondita
witzenbergiana
canaliculata
acuminata
sulphurea
odorata
glabra
nitida
lanceolata
nubigena
dracomontana
comptonii
rubropilosa
laetans
caffra
parvula
curvata
gaguedi
welwitschii
subulifolia
decurrens
cordata
amplexicaulis
lacticolor
mundii
aureaaurea
ureapotbergensi
mundiieast
subvestita
punctata
venusta
grandiceps
intonsa
tenax
montana
coronata
piscina
denticulata
scabra
neriifolia
laurifolia
pidocarpodendro
magnifica
longifolia
lorifolia
compacta
eximia
roupelliae
susannae
speciosa
pruinosa
cryophila
scolopendriifolia
cynaroides
-0.8
-0.4
0.0

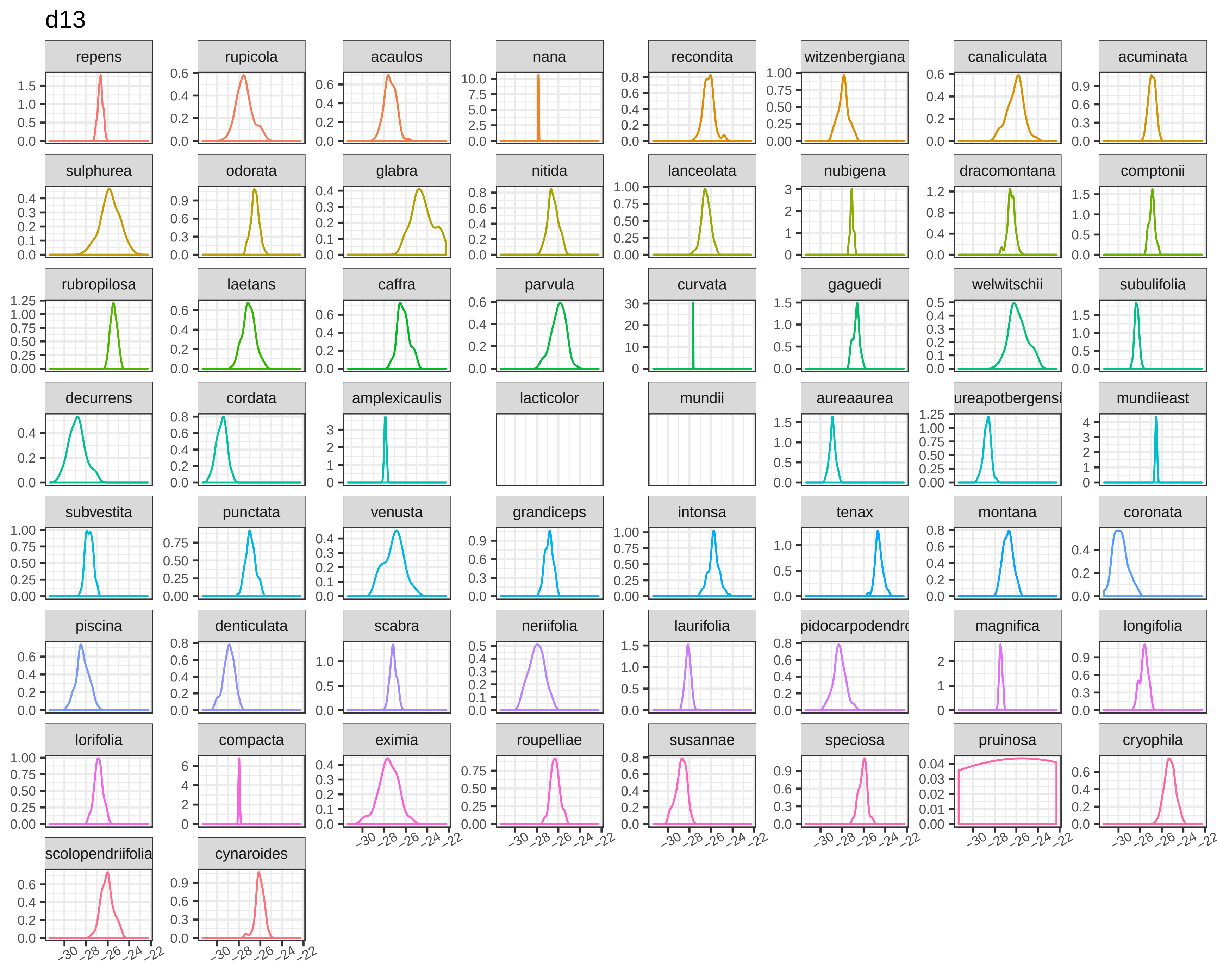

d13
repens
rupicola
acaulos
nana
recondita
witzenbergiana
canaliculata
acuminata
sulphurea
odorata
glabra
nitida
lanceolata
nubigena
dracomontana
comptonii
rubropilosa
laetans
caffra
parvula
curvata
gaguedi
welwitschii
subulifolia
decurrens
cordata
amplexicaulis
lacticolor
mundii
aureaaurea
ureapotbergensi
mundiieast
subvestita
punctata
venusta
grandiceps
intonsa
tenax
montana
coronata
piscina
denticulata
scabra
neriifolia
laurifolia
pidocarpodendro
magnifica
longifolia
lorifolia
compacta
eximia
roupelliae
susannae
speciosa
pruinosa
cryophila
scolopendriifolia
cynaroides
-30 -28 -26 -24 -22

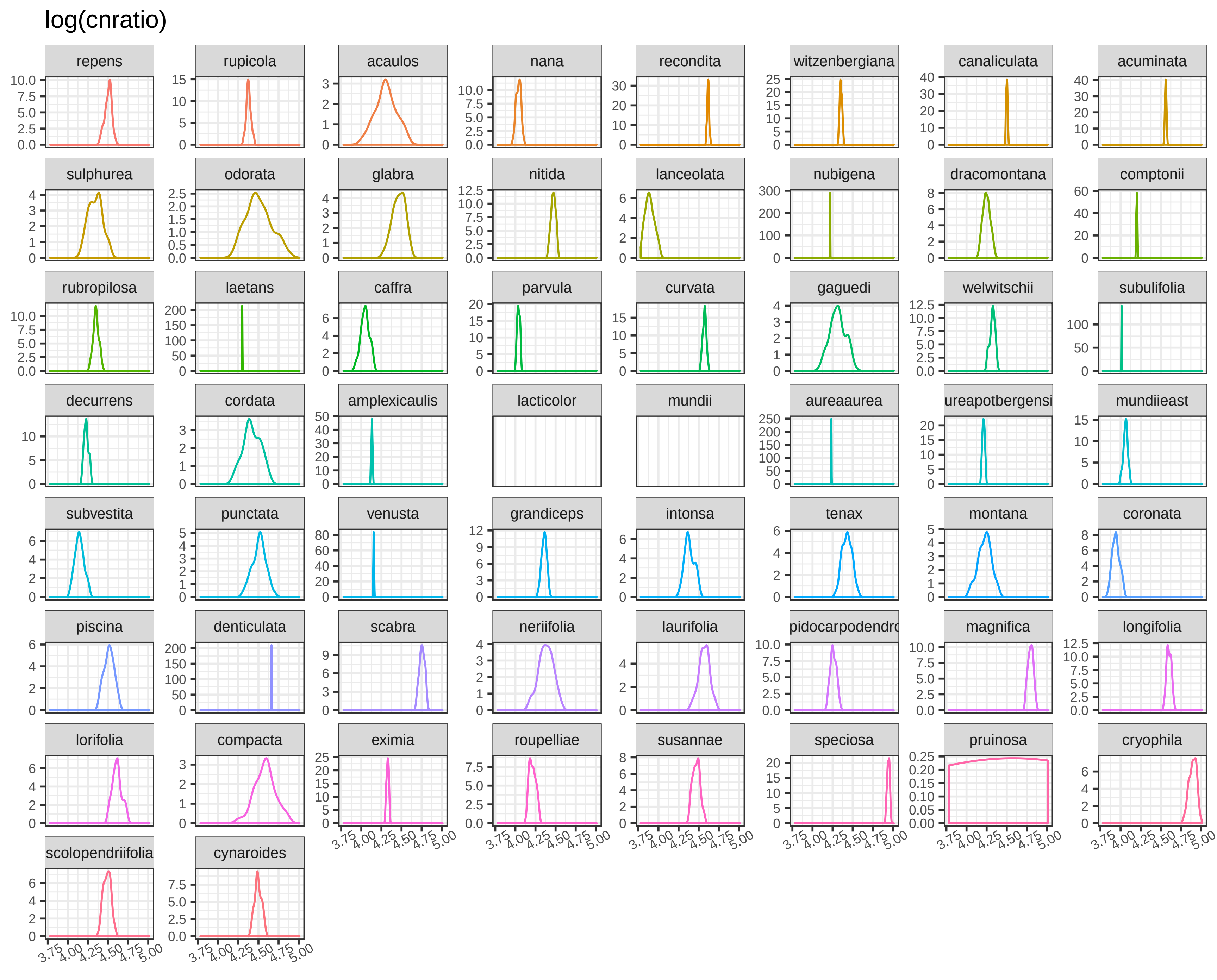
log(cnratio)
repens
rupicola
acaulos
nana
recondita
witzenbergiana
canaliculata
acuminata
sulphurea
odorata
glabra
nitida
lanceolata
nubigena
dracomontana
comptonii
rubropilosa
laetans
caffra
parvula
curvata
gaguedi
welwitschii
subulifolia
decurrens
cordata
amplexicaulis
lacticolor
mundii
aureaaurea
ureapotbergensi
mundiieast
subvestita
punctata
venusta
grandiceps
intonsa
tenax
montana
coronata
piscina
denticulata
scabra
neriifolia
laurifolia
pidocarpodendro
magnifica
longifolia
lorifolia
compacta
eximia
roupelliae
susannae
speciosa
pruinosa
cryophila
scolopendriifolia
cynaroides
3.75 4.00 4.25 4.50 4.75 5.00

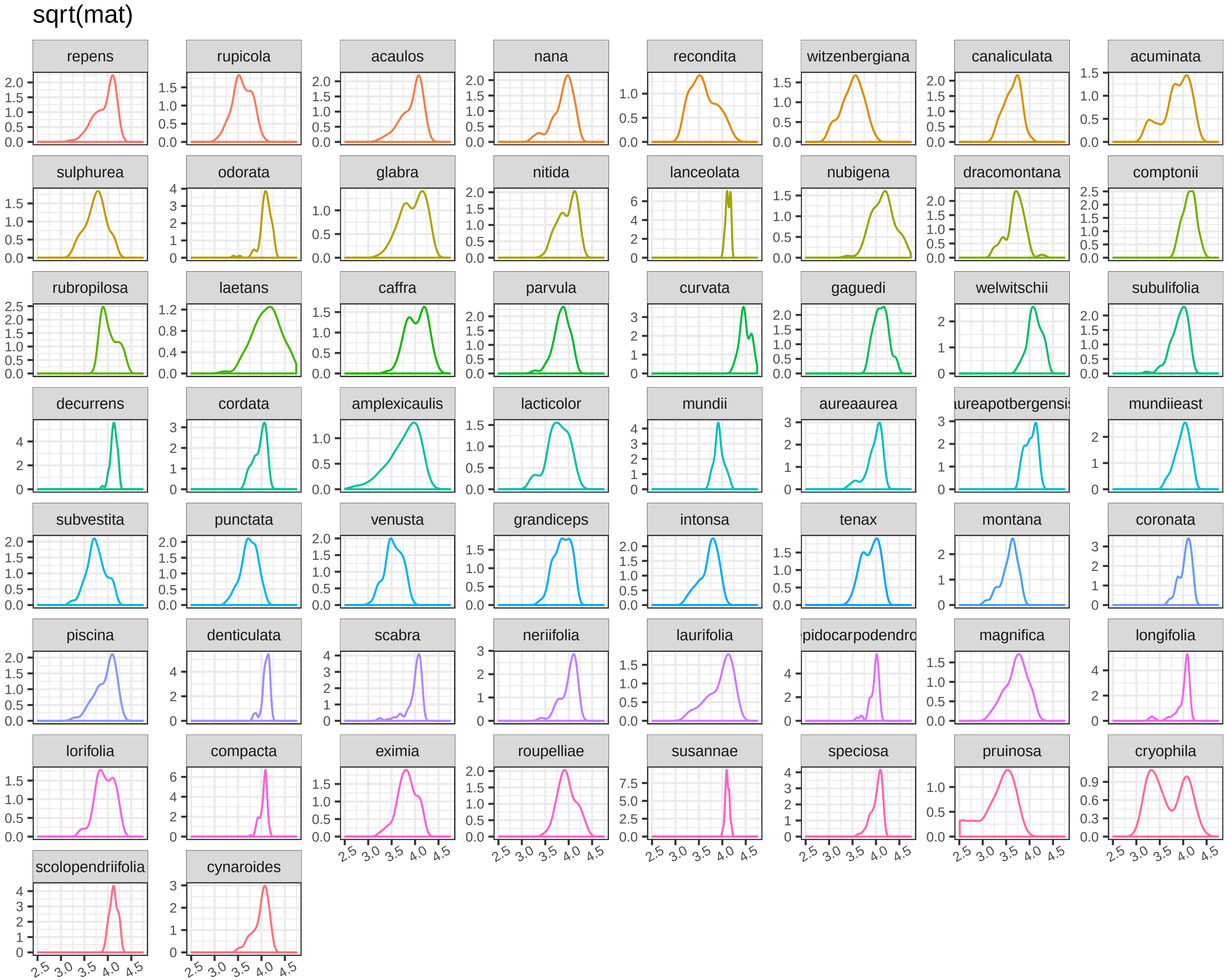

sqrt(mat)
repens
rupicola
acaulos
nana
recondita
witzenbergiana
canaliculata
acuminata
sulphurea
odorata
glabra
nitida
lanceolata
nubigena
dracomontana
comptonii
rubropilosa
laetans
caffra
parvula
curvata
gaguedi
welwitschii
subulifolia
decurrens
cordata
amplexicaulis
lacticolor
mundii
aureaaurea
ureapotbergensi
mundiieast
subvestita
punctata
venusta
grandiceps
intonsa
tenax
montana
coronata
piscina
denticulata
scabra
neriifolia
laurifolia
pidocarpodendro
magnifica
longifolia
lorifolia
compacta
eximia
roupelliae
susannae
speciosa
pruinosa
cryophila
scolopendriifolia
cynaroides
2.5 3.0 3.5 4.0 4.5

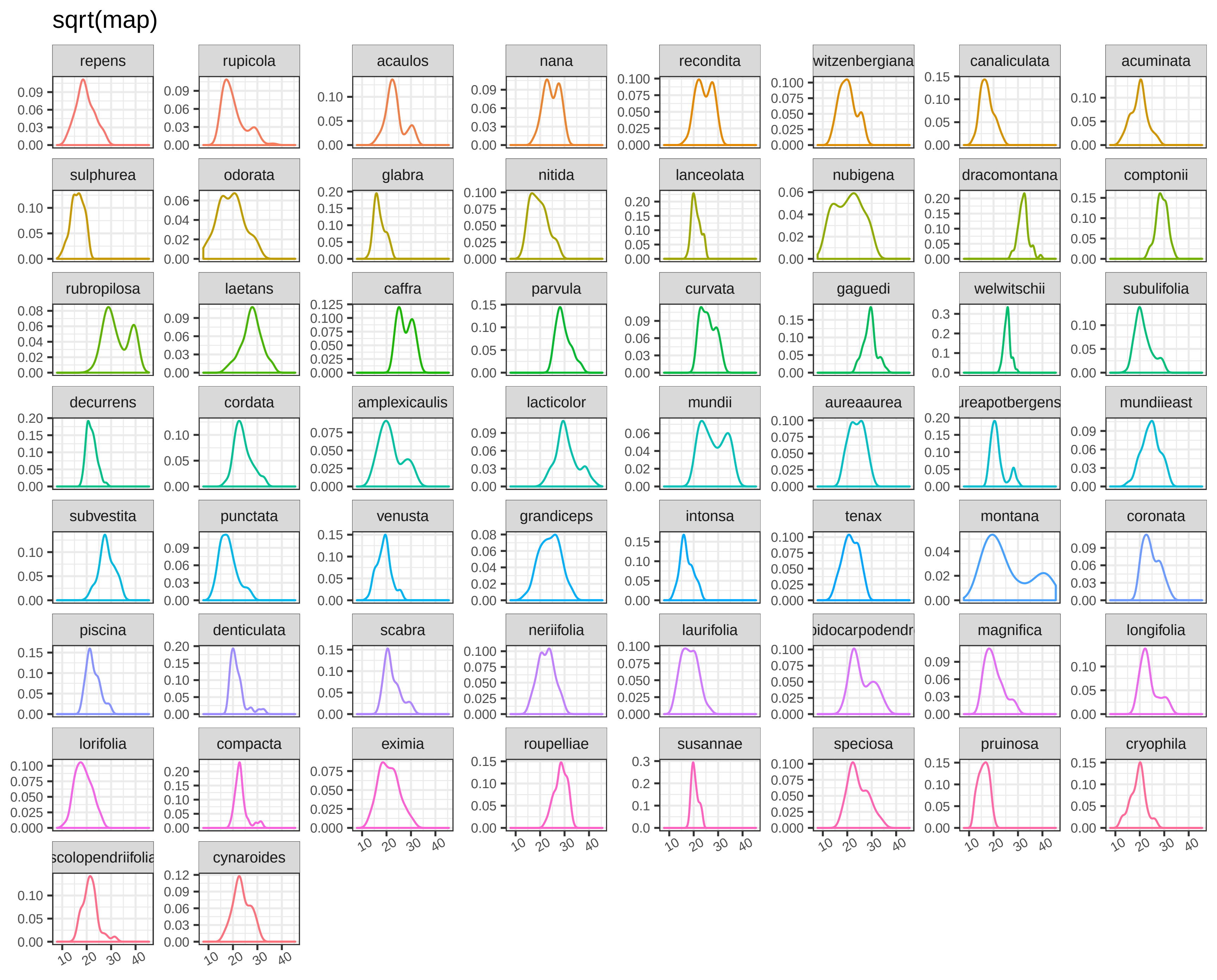

sqrt(map)
repens
rupicola
acaulos
nana
recondita
witzenbergiana
canaliculata
acuminata
sulphurea
odorata
glabra
nitida
lanceolata
nubigena
dracomontana
comptonii
rubropilosa
laetans
caffra
parvula
curvata
gaguedi
welwitschii
subulifolia
decurrens
cordata
amplexicaulis
lacticolor
mundii
aureaaurea
mundiieast
subvestita
punctata
venusta
grandiceps
intonsa
tenax
montana
coronata
piscina
denticulata
scabra
neriifolia
laurifolia
magnifica
longifolia
lorifolia
compacta
eximia
roupelliae
susannae
speciosa
pruinosa
cryophila
cynaroides
10
20
30
40

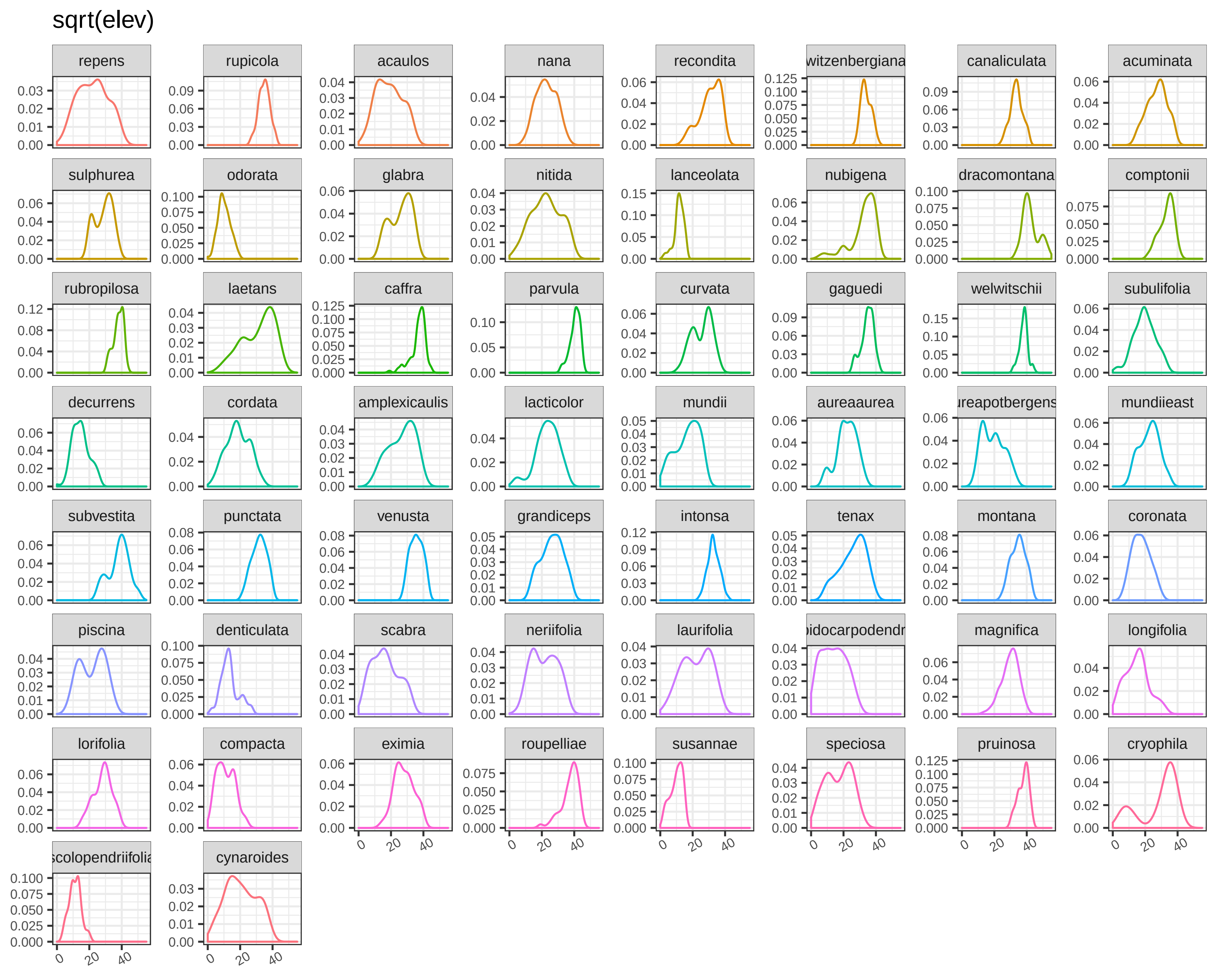

sqrt(elev)
repens
rupicola
acaulos
nana
recondita
witzenbergiana
canaliculata
acuminata
sulphurea
odorata
glabra
nitida
lanceolata
nubigena
dracomontana
comptonii
rubropilosa
laetans
caffra
parvula
curvata
gaguedi
welwitschii
subulifolia
decurrens
cordata
amplexicaulis
lacticolor
mundii
aureaaurea
ureapotbergens
mundiieast
subvestita
punctata
venusta
grandiceps
intonsa
tenax
montana
coronata
piscina
denticulata
scabra
neriifolia
laurifolia
idocarpodendr
magnifica
longifolia
lorifolia
compacta
eximia
roupelliae
susannae
speciosa
pruinosa
cryophila
scolopendriifolia
cynaroides
0
20
40

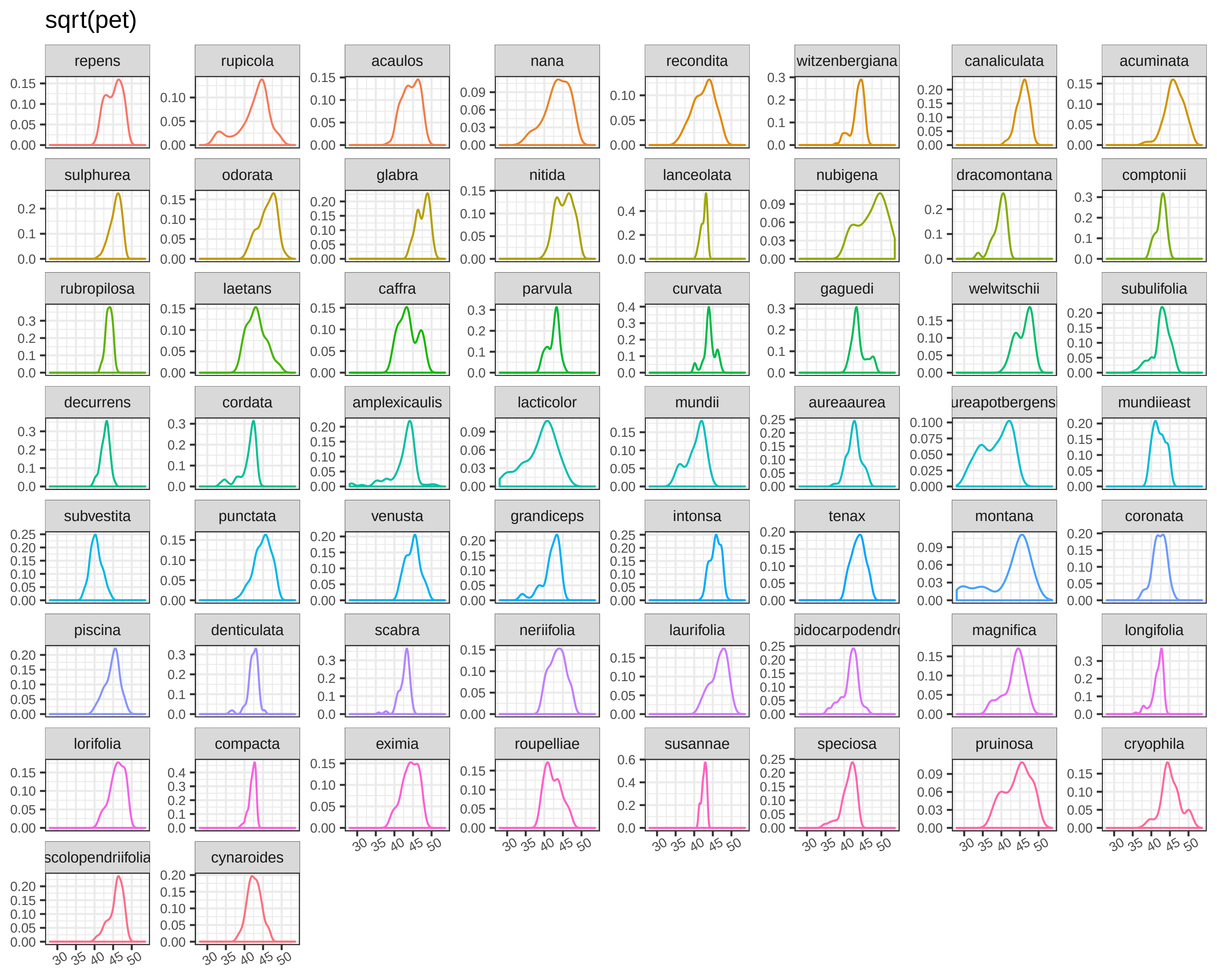
sqrt(pet)
repens
rupicola
acaulos
nana
recondita
witzenbergiana
canaliculata
acuminata
sulphurea
odorata
glabra
nitida
lanceolata
nubigena
dracomontana
comptonii
rubropilosa
laetans
caffra
parvula
curvata
gaguedi
welwitschii
subulifolia
decurrens
cordata
amplexicaulis
lacticolor
mundii
aureaaurea
ureapotbergens
mundiieast
subvestita
punctata
venusta
grandiceps
intonsa
tenax
montana
coronata
piscina
denticulata
scabra
neriifolia
laurifolia
idocarpodendr
magnifica
longifolia
lorifolia
compacta
eximia
roupelliae
susannae
speciosa
pruinosa
cryophila
scolopendriifolia
cynaroides
30 35 40 45 50

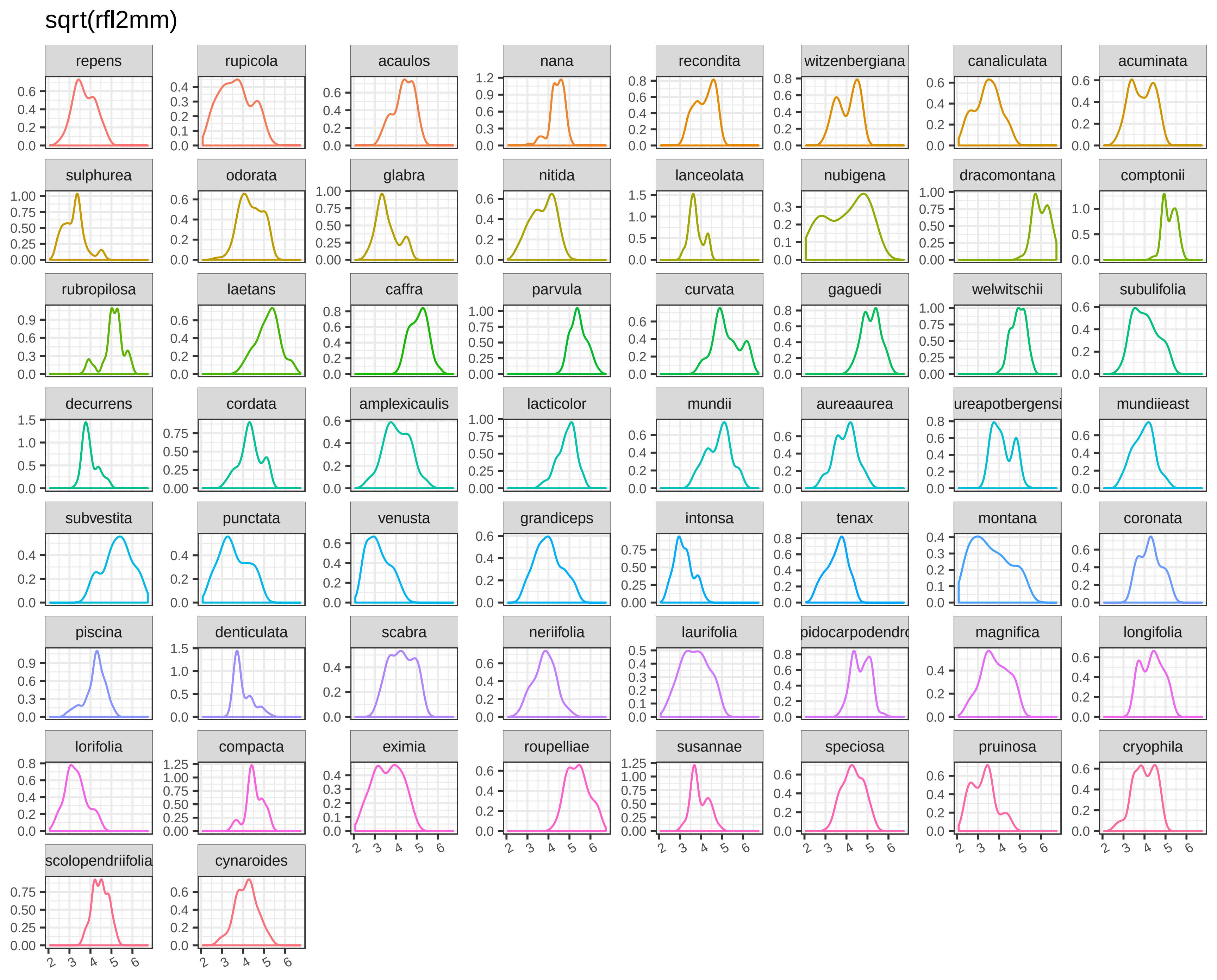
sqrt(rfl2mm)
repens
rupicola
acaulos
nana
recondita
witzenbergiana
canaliculata
acuminata
sulphurea
odorata
glabra
nitida
lanceolata
nubigena
dracomontana
comptonii
rubropilosa
laetans
caffra
parvula
curvata
gaguedi
welwitschii
subulifolia
decurrens
cordata
amplexicaulis
lacticolor
mundii
aureaaurea
ureapotbergensi
mundiieast
subvestita
punctata
venusta
grandiceps
intonsa
tenax
montana
coronata
piscina
denticulata
scabra
neriifolia
laurifolia
pidocarpodendro
magnifica
longifolia
lorifolia
compacta
eximia
roupelliae
susannae
speciosa
pruinosa
cryophila
scolopendriifolia
cynaroides

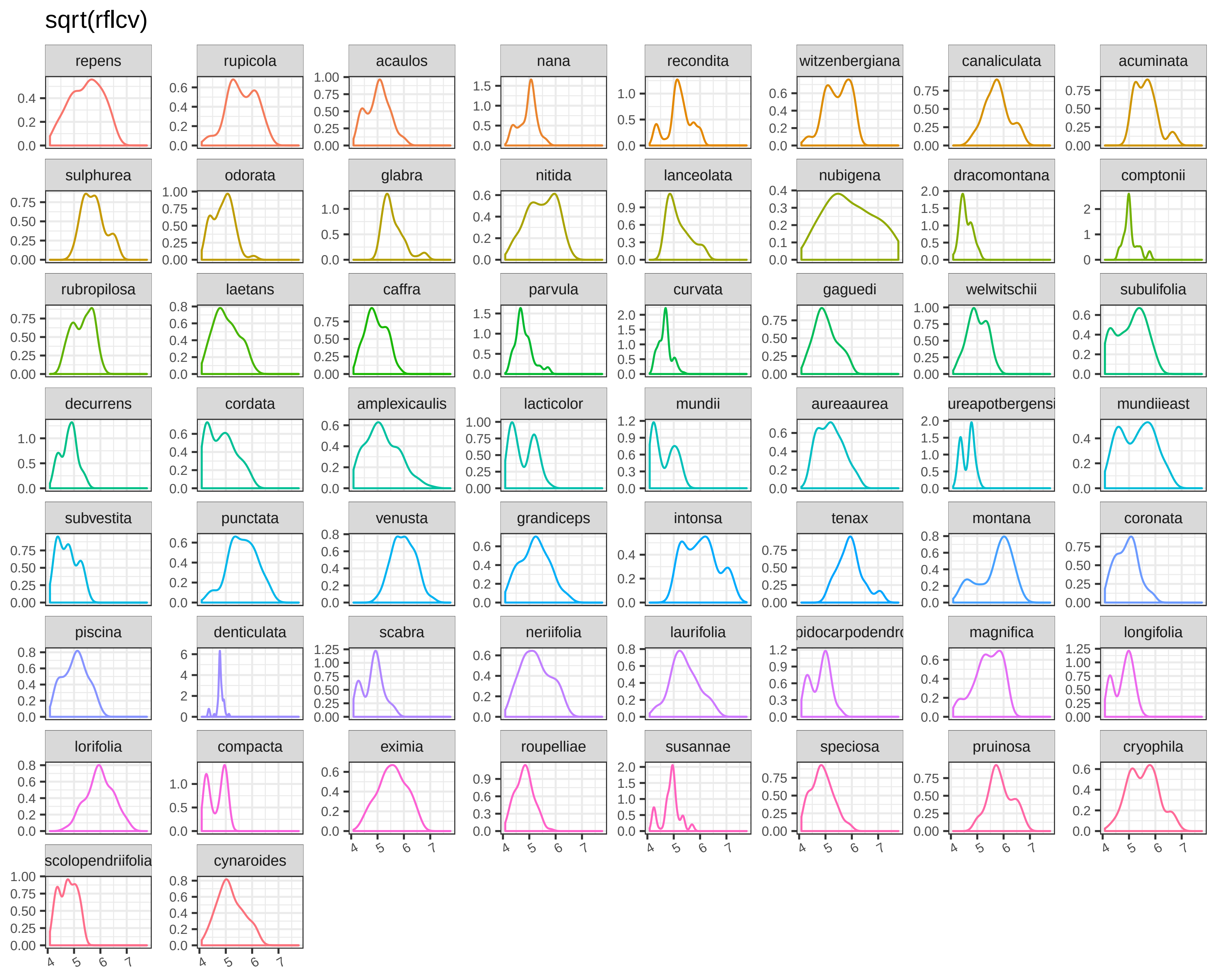
sqrt(rflcv)
repens
rupicola
acaulos
nana
recondita
witzenbergiana
canaliculata
acuminata
sulphurea
odorata
glabra
nitida
lanceolata
nubigena
dracomontana
comptonii
rubropilosa
laetans
caffra
parvula
curvata
gaguedi
welwitschii
subulifolia
decurrens
cordata
amplexicaulis
lacticolor
mundii
aureaaurea
ureapotbergensi
mundiieast
subvestita
punctata
venusta
grandiceps
intonsa
tenax
montana
coronata
piscina
denticulata
scabra
neriifolia
laurifolia
pidocarpodendro
magnifica
longifolia
lorifolia
compacta
eximia
roupelliae
susannae
speciosa
pruinosa
cryophila
scolopendriifolia
cynaroides
4
5
6
7

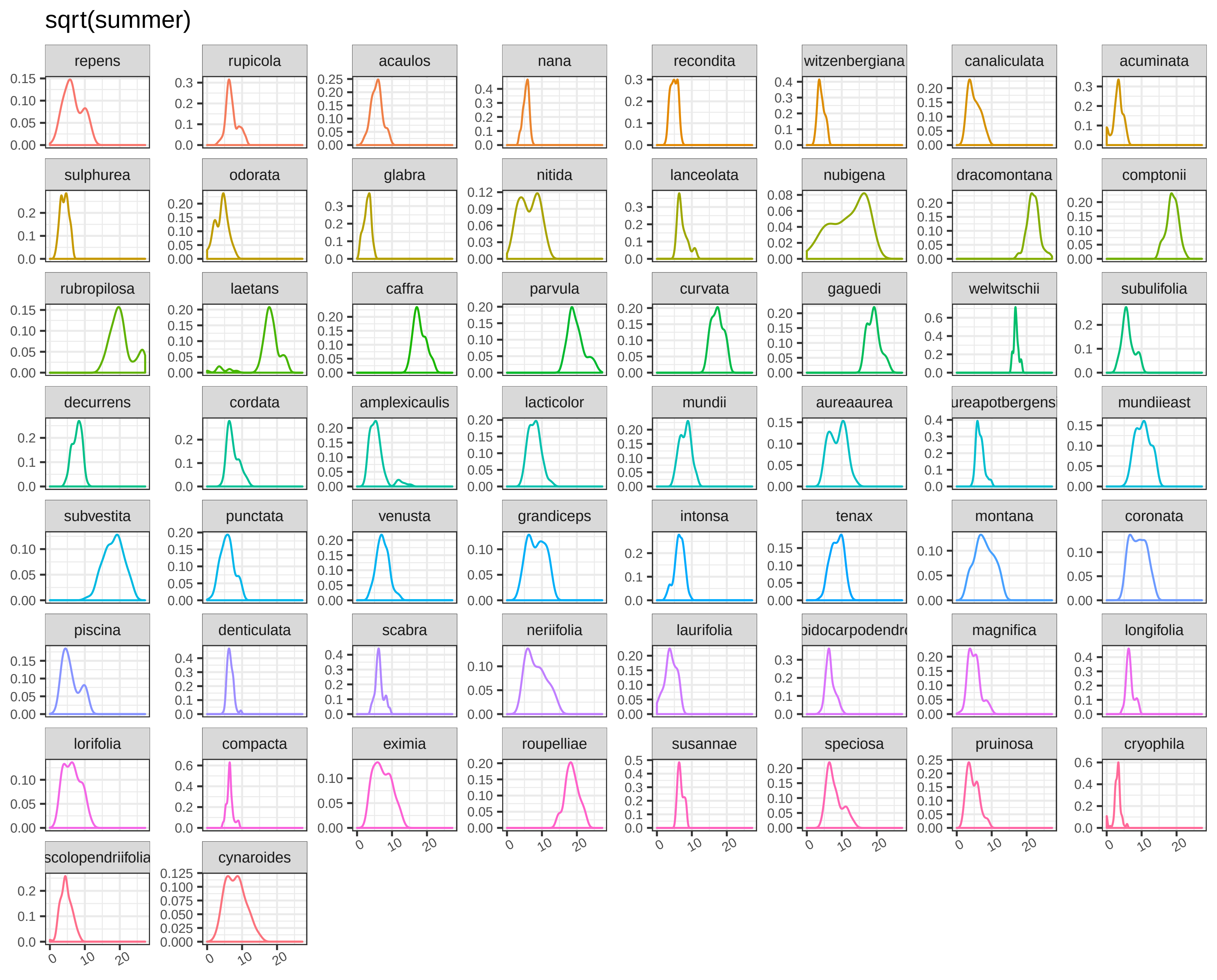
sqrt(summer)
repens
rupicola
acaulos
nana
recondita
witzenbergiana
canaliculata
acuminata
sulphurea
odorata
glabra
nitida
lanceolata
nubigena
dracomontana
comptonii
rubropilosa
laetans
caffra
parvula
curvata
gaguedi
welwitschii
subulifolia
decurrens
cordata
amplexicaulis
lacticolor
mundii
aureaaurea
ureapotbergens
mundiieast
subvestita
punctata
venusta
grandiceps
intonsa
tenax
montana
coronata
piscina
denticulata
scabra
neriifolia
laurifolia
pidocarpodendr
magnifica
longifolia
lorifolia
compacta
eximia
roupelliae
susannae
speciosa
pruinosa
cryophila
scolopendriifolia
cynaroides

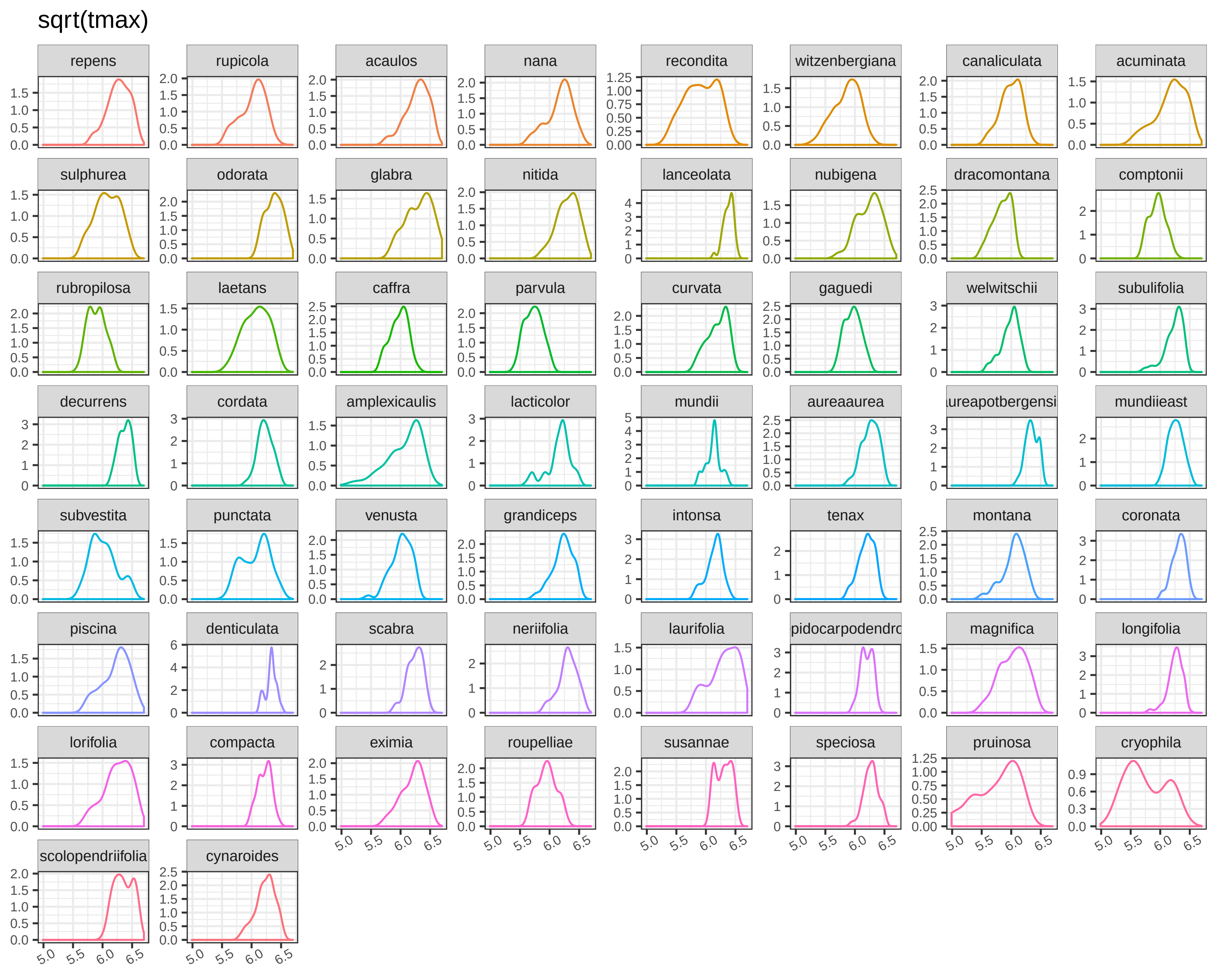

sqrt(tmax)
repens
rupicola
acaulos
nana
recondita
witzenbergiana
canaliculata
acuminata
sulphurea
odorata
glabra
nitida
lanceolata
nubigena
dracomontana
comptonii
rubropilosa
laetans
caffra
parvula
curvata
gaguedi
welwitschii
subulifolia
decurrens
cordata
amplexicaulis
lacticolor
mundii
aureaaurea
ureapotbergensi
mundiieast
subvestita
punctata
venusta
grandiceps
intonsa
tenax
montana
coronata
piscina
denticulata
scabra
neriifolia
laurifolia
pidocarpodendro
magnifica
longifolia
lorifolia
compacta
eximia
roupelliae
susannae
speciosa
pruinosa
cryophila
scolopendriifolia
cynaroides
5.0
5.5
6.0
6.5

(tmin)

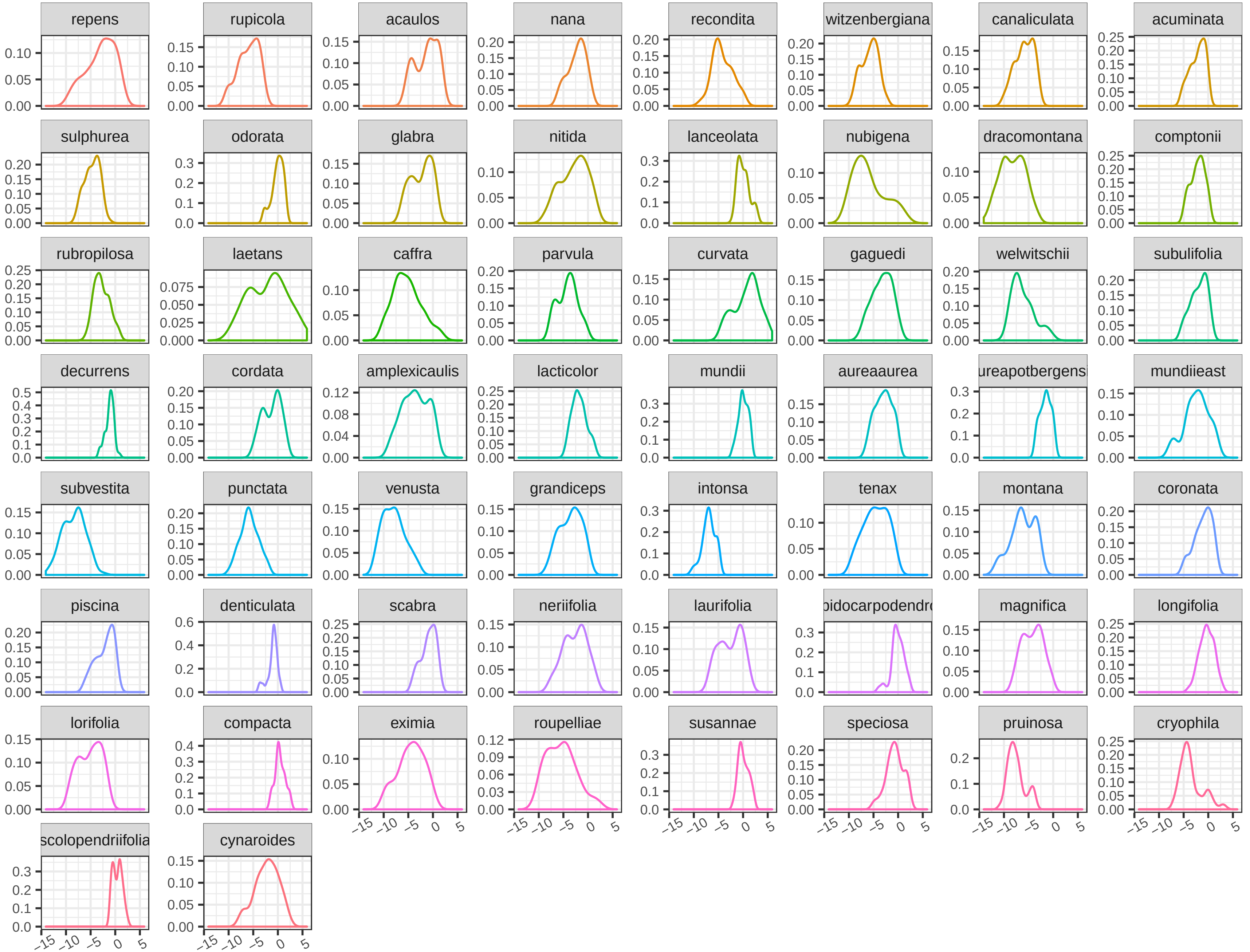
repens
rupicola
acaulos
nana
recondita
witzenbergiana
canaliculata
acuminata
sulphurea
odorata
glabra
nitida
lanceolata
nubigena
dracomontana
comptonii
rubropilosa
laetans
caffra
parvula
curvata
gaguedi
welwitschii
subulifolia
decurrens
cordata
amplexicaulis
lacticolor
mundii
aureaaurea
mundiieast
subvestita
punctata
venusta
grandiceps
intonsa
tenax
montana
coronata
piscina
denticulata
scabra
neriifolia
laurifolia
magnifica
longifolia
lorifolia
compacta
eximia
roupelliae
susannae
speciosa
pruinosa
cryophila
scolopendriifolia
cynaroides
-15 -10 -5 0 5

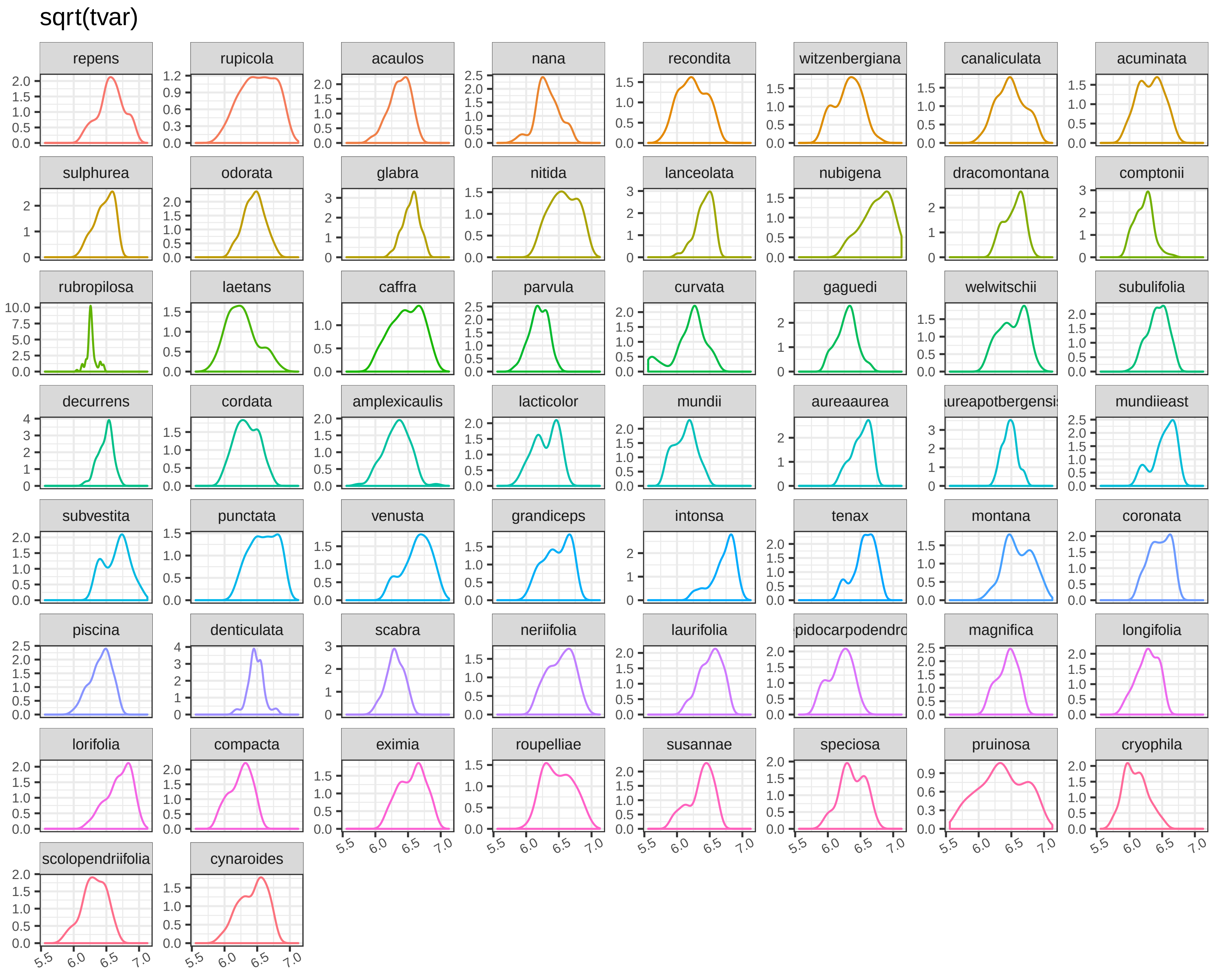
sqrt(tvar)
repens
rupicola
acaulos
nana
recondita
witzenbergiana
canaliculata
acuminata
sulphurea
odorata
glabra
nitida
lanceolata
nubigena
dracomontana
comptonii
rubropilosa
laetans
caffra
parvula
curvata
gaguedi
welwitschii
subulifolia
decurrens
cordata
amplexicaulis
lacticolor
mundii
aureaaurea
ureapotbergensis
mundiieast
subvestita
punctata
venusta
grandiceps
intonsa
tenax
montana
coronata
piscina
denticulata
scabra
neriifolia
laurifolia
pidocarpodendro
magnifica
longifolia
lorifolia
compacta
eximia
roupelliae
susannae
speciosa
pruinosa
cryophila
scolopendriifolia
cynaroides
5.5
6.0
6.5
7.0
