# Supplementary figures and images for: Correlated evolution between climate and suites of traits along a fast–slow continuum in the radiation of Protea
Source: Ecol Evol. 2018 Jan 12;8(3):1853–66. doi: 10.1002/ece3.3773 (PMC5792567; doi:10.1002/ece3.3773)

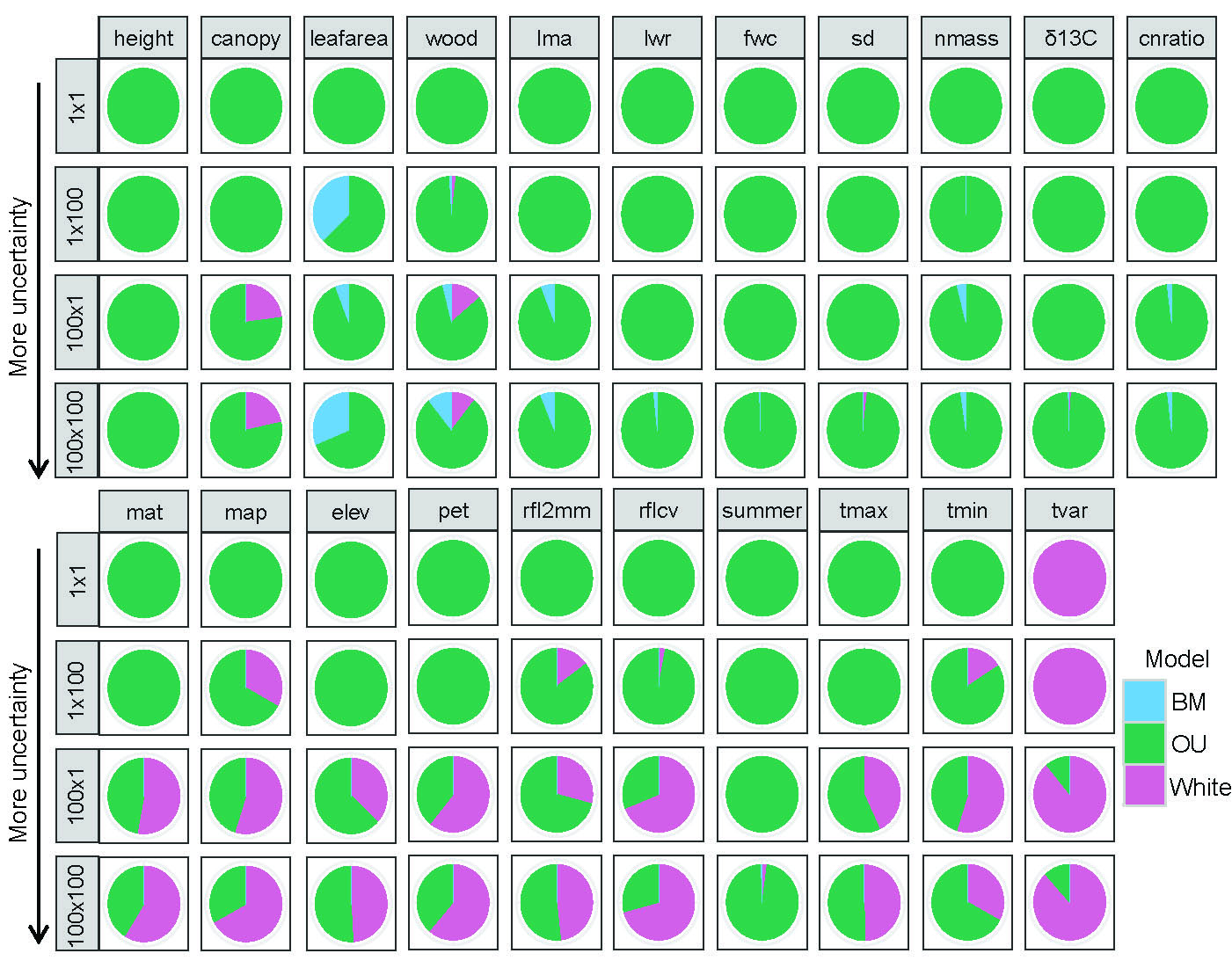

Supplement: Supplementary file 2 [file ECE3-8-1853-s002.tif]
